# Immunological design of commensal communities to treat intestinal infection and inflammation

**DOI:** 10.1371/journal.ppat.1009191

**Published:** 2021-01-19

**Authors:** Rebecca L. Brown, Max L. Y. Larkinson, Thomas B. Clarke

**Affiliations:** MRC Centre for Molecular Bacteriology and Infection, Department of Infectious Disease, Imperial College London, London, United Kingdom; McMaster University, CANADA

## Abstract

The immunological impact of individual commensal species within the microbiota is poorly understood limiting the use of commensals to treat disease. Here, we systematically profile the immunological fingerprint of commensals from the major phyla in the human intestine (Actinobacteria, Bacteroidetes, Firmicutes and Proteobacteria) to reveal taxonomic patterns in immune activation and use this information to rationally design commensal communities to enhance antibacterial defenses and combat intestinal inflammation. We reveal that Bacteroidetes and Firmicutes have distinct effects on intestinal immunity by differentially inducing primary and secondary response genes. Within these phyla, the immunostimulatory capacity of commensals from the Bacteroidia class (Bacteroidetes phyla) reflects their robustness of TLR4 activation and Bacteroidia communities rely solely on this receptor for their effects on intestinal immunity. By contrast, within the Clostridia class (Firmicutes phyla) it reflects the degree of TLR2 and TLR4 activation, and communities of Clostridia signal via both of these receptors to exert their effects on intestinal immunity. By analyzing the receptors, intracellular signaling components and transcription factors that are engaged by different commensal species, we identify canonical NF-κB signaling as a critical rheostat which grades the degree of immune stimulation commensals elicit. Guided by this immunological analysis, we constructed a cross-phylum consortium of commensals (*Bacteroides uniformis*, *Bacteroides ovatus*, *Peptostreptococcus anaerobius* and *Clostridium histolyticum*) which enhances innate TLR, IL6 and macrophages-dependent defenses against intestinal colonization by vancomycin resistant Enterococci, and fortifies mucosal barrier function during pathological intestinal inflammation through the same pathway. Critically, the setpoint of intestinal immunity established by this consortium is calibrated by canonical NF-κB signaling. Thus, by profiling the immunological impact of major human commensal species our work paves the way for rational microbiota reengineering to protect against antibiotic resistant infections and to treat intestinal inflammation.

## Author summary

The symbiotic bacteria that colonize the human intestine are major regulators of the immune system. While the collective ability of these organisms to shape immune function is clear the individual impact of different symbionts on immunity is poorly delineated. To address this, we have systematically characterized the immunological properties of an extensive panel of commensals from the major taxa in the human intestinal microbiota. We have uncovered the unique complement of pattern recognition receptors, intracellular signaling components and transcription factors through which each symbiotic species exerts their effect on the innate immune system. This allowed us to identify, for the first time, taxonomic patterns of commensal regulation of immunity and uncover a unique role for canonical NF-κB signaling in acting as a rheostat calibrating the degree of immune stimulation symbionts elicit. We used the immunological profiles of these symbionts to guide the construction of therapeutic symbiont communities to combat both AMR infection and pathological intestinal inflammation. Previously, harnessing the power of symbiotic microbes to combat disease was largely a trial-and-error process, and predicting how changes in microbiota composition will impact immunity was almost impossible. Our study addresses both of these challenges and provides a unique scientific resource to guide the rational design of symbiotic microbial communities to treat myriad diseases and reveals how loss of different symbionts will impact host health.

## Introduction

The microbiota is well established as a major regulator of immune development and functional maturation[1–4]. The most significant microbiota colonizing humans, in terms of microbial mass, species diversity and influence on immunity, is found within the intestine[5]. The intestinal microbiota is dominated by commensal bacteria from two phyla: the Firmicutes and the Bacteroidetes, with species from the Actinobacteria and Proteobacteria phyla comprising the majority of the remaining species[5,6]. While the collective ability of these organisms to regulate immunity has been thoroughly investigated, the individual contributions of different commensal species within the microbiota to the regulation of immunity is poorly understood[1,7,8]. Changes in the taxonomic composition of the microbiota (dysbiosis) are linked to a variety of diseases and dysfunctions, including acute and chronic inflammatory conditions, autoimmunity, and increased susceptibility to infection[2,4,5]. The underlying etiology of these conditions is thought to be driven by aberrant interactions between the immune system and dysbiotic microbiota indicating that not all members of the microbiota have an equivalent influence on immunity[2,8]. Thus, delineating the immunostimulatory properties of different commensal species will allow rational approaches to microbiota reengineering to combat disease.

The translation of microbial signals from the microbiota into changes in immune system behavior occurs principally through the pattern recognition receptors (PRRs) of the innate immune system[1,9,10]. Recognition of microbiota-derived products by PRRs occurs during homeostasis and has been shown to influence innate immunity both within the intestine and also at extra-intestinal sites[1,11–16]. This homeostatic PRR stimulation by the microbiota has a profound effect on the functioning of innate immune cells. It has now been demonstrated that nearly every part of the innate cell life-cycle (including production[14,17], migration[18], functional maturation[15] and death[19]) is under the influence of the microbiota through PRRs. The importance to PRR stimulation is not limited to homeostasis. During intestinal inflammation, PRR signaling promotes mucosal repair and the restoration of immune homeostasis[11], similarly PRR stimulation can also promote the clearance of pathogens from the intestine[20]. This highlights the potential utility of PRR stimulation by commensals to treat disease. Of the major families of these innate receptors, it is stimulation of Toll-like receptors (TLRs) that is central to converting signals from the bacteria in the microbiota into changes in innate immune cell function[9,10]. TLRs primarily recognize the immunostimulatory cell surface molecules of bacteria and it is these microbial products, such as lipopolysaccharide, peptidoglycan, lipoproteins and lipoteichoic acids that are responsible for programming our immune system from birth[21,22]. Despite being highly conserved molecules produced by the preponderance of bacteria, each commensal species is likely to produce a different complement of these molecules, in different amounts, and of different intrinsic immunological potencies. Thus, each commensal will have a unique “immunological fingerprint” that will determine it’s influence on the immune system. Given the spectrum and complexity of the biosynthesis of these immunologically active molecules the degree of immune stimulation exerted by a particular commensal species is therefore extremely difficult to predict from genome analysis alone. Thus, despite the intestinal microbiota comprising hundreds of commensal species there are only a very limited number of commensals whose specific impact on immunity is thoroughly understood[7,8,23]. It is currently unclear, therefore, whether microbiota-mediated regulation of immunity relies on a small cadre of keystone microbial taxa that act as central regulators of immunity, or whether there is redundancy between commensals in controlling immunity due to shared immunological properties within commensal taxa[1,8,24]. This raises a central problem in our current understanding of the microbiota: given the vastness of the microbiota, how do you begin to identify commensals that could therefore be used therapeutically to treat disease?

To address this challenge, here, we analyzed the immunostimulatory capacity of a broad panel of commensals from the major bacterial phyla in the human intestinal microbiota. By systemically characterizing the TLRs, intracellular signaling components and transcription factors which convert cues from these commensal into changes in host immunity, we identify taxonomic patterns in the effects of commensals on innate immunity. We used this immunological information to rationally design commensal consortia which enhance intestinal immune defenses against colonization by antibiotic resistant pathogens and also restore intestinal homeostasis during pathological inflammation. Ultimately, we find that canonical NF-κB signaling is the critical host factor acting as a rheostat to determine the impact of commensals on immune functioning.

## Results

### Identification of potent immunostimulatory commensals from the human microbiota

**Fig 1:**
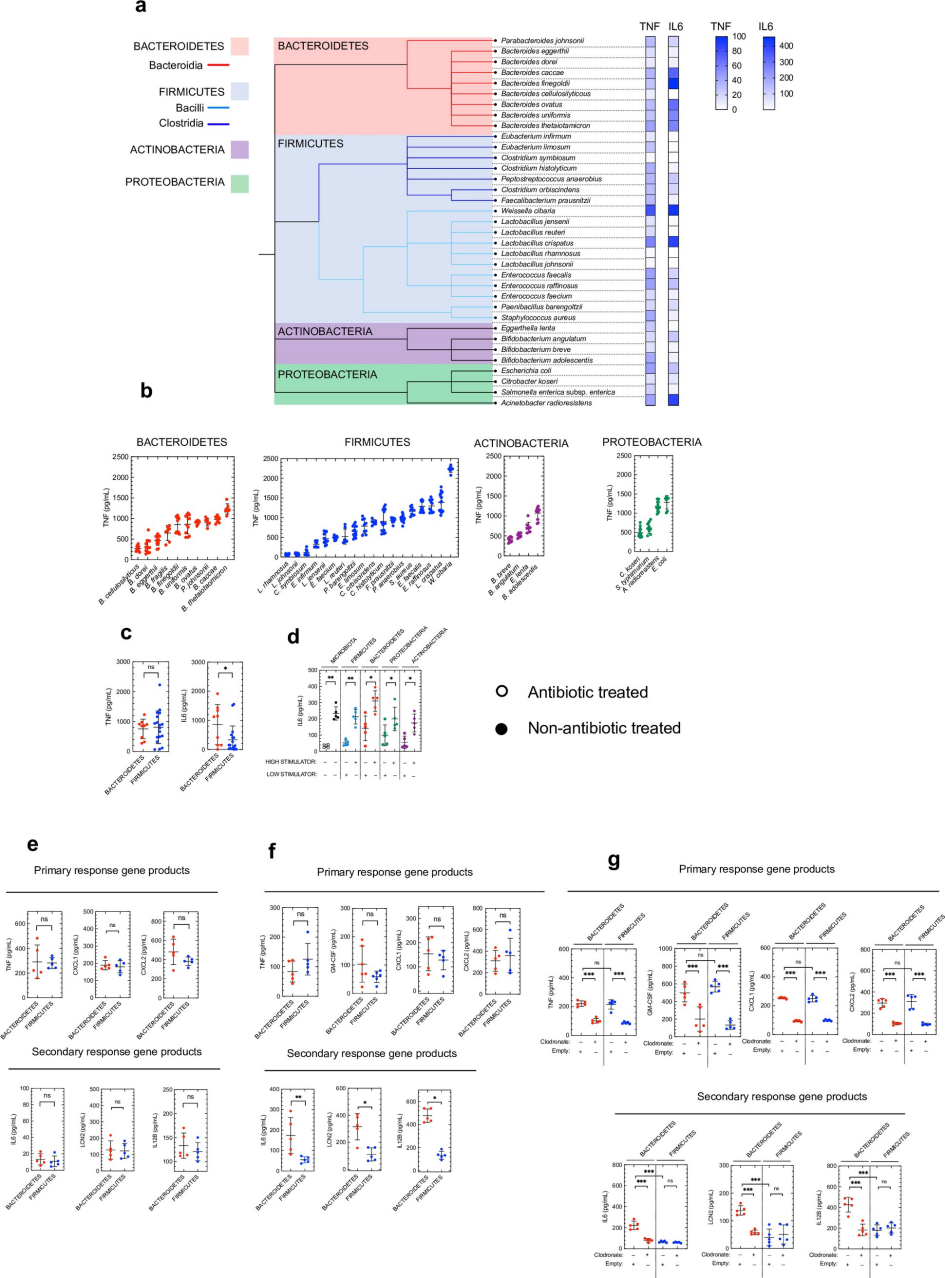
Identification of commensal microbes that are robust activators of innate immunity. (a) Cladogram of intestinal commensal species used in this study. Heat map displaying fold induction in macrophage TNF and IL6 production relative to unstimulated cells (mean values for each species are shown, n = 6–15 biological repeats, at MOI of 1:10). (b) Absolute levels of TNF produced by each commensal species ranked from low to high stimulators within each phyla. (c) Comparison of average induction of TNF and IL6 by members of the Bacteroidetes and Firmicute phyla. Each data point is mean level of cytokine production for a single commensal species, horizontal lines indicate median values. (d) Intestinal IL6 levels 24 hours post oral inoculation with 1×10^8^ CFU indicated commensal species. Each point represents a single mouse and horizontal lines indicate median values (HIGH STIMULATORS: Actinobacteria: *Bifidobacterium adolescentis*; Bacteroidetes: *Bacteroides caccae*; Firmicutes: *Eubacterium infirmum*; Proteobacteria: *Acinetobacter radioresistens*) (LOW STIMULATORS: Actinobacteria: *Bifidobacterium breve*; Bacteroidetes: *Bacteroides cellulosilyticus*; Firmicutes: *Enterococcus raffinosus*; Proteobacteria: *Citrobacter koseri*). All mice were antibiotic treated unless indicated otherwise prior to commensal administration. (e,f,g) Intestinal cytokine levels 4 (e) and 24 (f,g) hours post oral inoculation with 1×10^8^ CFU of indicated commensal consortium (BACTEROIDETES consortium: *Bacteroides caccae*, *Bacteroides cellulosilyticus*, *Bacteroides dorei*, *Bacteroides eggerthii*, *Bacteroides finegoldii*, *Bacteroides ovatus*, *Bacteroides thetaiotaomicron*, *Bacteroides uniformis*, *Parabacteroides johnsonii*; FIRMICUTES consortium: *Clostridium histolyticum*, *Clostridium orbiscindens*, *Enterococcus faecalis*, *Enterococcus raffinosus*, *Eubacterium limosum*, *Faecalibacterium prausnitzii*, *Lactobacillus reuteri*, *Lactobacillus crispatus*, *Paenibacillus barengoltzii*, *Peptostreptococcus anaerobius*). For (g) indicated mice were treated with clodronate liposomes or empty liposomes 2 days prior and concomitant with consortia inoculation. All mice were antibiotic treated unless indicated otherwise prior to commensal administration. Horizontal lines indicate mean values (d-g) and statistical comparisons were by Mann Whitney test *p < 0.05, **p < 0.01, and NS, not significant.

To understand the impact of different commensal taxa on immunity, we cultured a broad panel of organisms from the major phyla from the human intestinal microbiota: the Bacteroidetes, Firmicutes, Actinobacteria and Proteobacteria[6]. We assembled a panel of 35 bacteria, the majority (27/35) from the dominant two phyla within the intestinal microbiota: Firmicutes and Bacteroidetes (Fig 1A). Our panel of commensals included members of three of the most common bacterial classes in the intestinal microbiota: Clostridia (Firmicutes), Bacilli (Firmicutes), and Bacteroidia (Bacteroidetes)[6]. We wanted to understand how these commensals influence innate immunity as this arm of the immune system is heavily influence by commensal bacteria[25]. To begin to understand the immunostimulatory capacity of our commensal panel, we stimulated the macrophage-like cell line (J774A.1) with each commensal individually and measured the production of the cytokines IL6 and TNF. We chose macrophages as a model because *in vivo* these cells are heavily influenced by signals from the microbiota[26], they are positioned within tissues at the interface with the microbiota[27], and express a broad complement of PRRs[28]. We measured IL6 and TNF as the production of these cytokines in macrophages relies on PRRs[29,30] and can therefore be used as a “readout” of commensal-induced innate immune activation (hereafter referred to as “immunostimulation”); IL6 and TNF are critical host signals in the intestine that mediate many of the effects of the microbiota on the immune system[27,31,32]; and they are products of primary (*Tnf*) and secondary (*Il6*) response genes[29,30,33], thus relying on different mechanisms to control their production. To begin our analysis of this, we used heat-killed bacteria to separate the impact of structural PRR ligands produced by each commensal, which are insensitive to heat treatment, from the metabolic products of commensals.

Across the panel as a whole, we found that there was significant variation in the immunostimulatory activity of commensals, as measured by the levels of TNF and IL6 they induce in macrophages (Fig 1A and 1B). This variation was evident between commensal species within the major phyla Bacteroidetes and Firmicutes. For example, *Bacteroides caccae* (Bacteroidetes) and *Lactobacillus crispatus* (Firmicutes) were both potent innate immune activators. *Bacteroides caccae* induced an 35-fold increase in TNF and 332-fold increase in IL6, above unstimulated controls. Similarly, *Lactobacillus crispatus* induced a 58-fold increase in TNF and 401-fold increase in IL6. By contrast, *Bacteroides cellulosilyticus* (Bacteroidetes) and *Clostridium symbiosum* (Firmicutes) both induced weak immune stimulators. *Bacteroides cellulosilyticus* induced an 9-fold increase in TNF and 0.4-fold increase in IL6, above unstimulated controls. Similarly, *Clostridium symbiosum* induced a 4-fold increase in TNF and 0.1-fold increase in IL6. Our screen of commensal-driven innate immune activation was performed with a macrophage-like cell line (J774A.1), next, therefore, we used bone marrow-derived macrophages (BMDMs) to confirm that the results from our cell line were broadly consistent between cells and thus a reliable reflection of the immunostimulatory capacities of our commensal panel. We stimulated BMDMs with a number of our commensal species and measured cytokine production. We found a strong correlation between the level of cytokine production after commensal stimulation of our cell line and BMDMs (S1 Fig), supporting the notion that cytokine production by our macrophage cell line is a fair reflection of the immunostimulatory capacity of our commensal panel. As both the J774A.1 cells and bone marrow-derived macrophages are murine cells, we wanted to ensure that our commensals elicited a similar pattern of immune activation in human cells too. To do this we stimulated differentiated THP-1 cells with members of our commensal panel. Using this human macrophage-like cell line, we found a strong correlation between the levels of cytokine elicited by commensals stimulation from these cells and our murine cell line (S2 Fig) indicating that our murine models were relevant to humans and that the commensal species in our panel are likely to have a similar effect on human and mouse cells. Finally, to ensure that any differences in cytokine production were not due to the differential induction of cell death in macrophages by commensals, we measured lactate dehydrogenase release by macrophages after commensal stimulation. We found that none of our commensals induced macrophage cell death confirming that any differences in cytokine induction was solely down to differences in the immunostimulatory capacity of each species (S3 Fig).

### Bacteroidetes and Firmicutes have differential effects on primary and secondary responses genes in the intestine

Next, we wanted to understand whether there were any trends in innate immune activation that reflected the taxonomy of our commensal panel. To investigate whether there was any difference in immunostimulatory capacity between the dominant intestinal phyla, we compared mean cytokine production induced by each Bacteroidetes species with the mean cytokine production elicited by each Firmicute species. We found no significant difference in the levels of TNF induced by Bacteroidetes and Firmicutes (Fig 1C). By contrast, we found that commensal Bacteroidetes tended to induce significantly more IL6 than the Firmicutes (Fig 1C). This indicates that, as a whole, there is no distinction in the ability of Bacteroidetes and Firmicutes to induce cytokines that are the products of primary response genes, however, Bacteroidetes tend to be better at inducing the production cytokines from secondary response genes.

**Fig 2:**
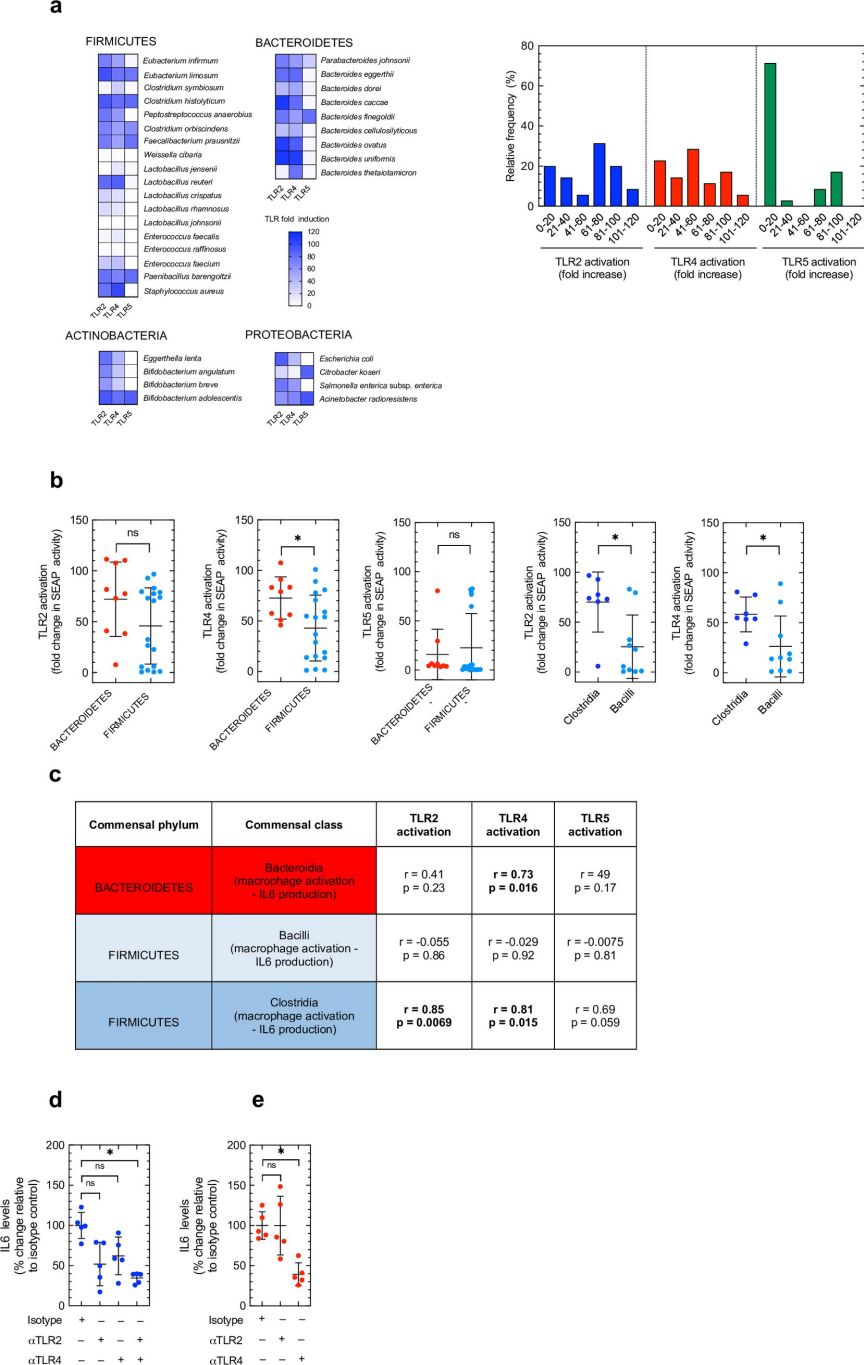
Patterns of commensal immunostimulation within the Bacteroidetes and Firmicutes phyla reflect and rely upon the activation of different TLRs. (a) TLR-dependent SEAP production by HEK293 cells 24 hours post-stimulation with indicated commensal species (at MOI of 1:10). Corresponding frequency distribution of TLR-dependent SEAP production for all commensals in our panel. (b) Comparison of TLR activation by members of the Bacteroidetes and Firmicute phyla. Each data point is mean fold increase in TLR-dependent SEAP activity elicited by a single commensal species, horizontal lines indicate median values. (c) Correlation between overall immunostimulatory capacity of commensals in indicated phyla (measured by macrophage IL6 production (using data from Fig 1A)) and TLR-dependent SEAP activation. Correlations were determined using the Pearson’s test. (d,e) Intestinal cytokine levels 24 hours post oral inoculation with 1×10^8^ CFU of indicated commensal consortium ((d) Clostridia consortium: *Eubacterium infirnum*, *Clostridium histolyticum*, *Clostridium orbiscindens*, *Clostridium symbiosum*, *Eubacterium limosum*, *Faecalibacterium prausnitzii*, *Peptostreptococcus anaerobius*. (e) Bacteroidia consortium: *Bacteroides caccae*, *Bacteroides cellulosilyticus*, *Bacteroides dorei*, *Bacteroides eggerthii*, *Bacteroides finegoldii*, *Bacteroides ovatus*, *Bacteroides thetaiotaomicron*, *Bacteroides uniformis*). Indicated groups of mice were treated with TLR neutralizing antibodies, or isotype control, (100 μg/mouse) concomitant with consortia inoculation. All mice were antibiotic treated unless indicated otherwise prior to commensal administration. Each point represents a single mouse and horizontal lines indicate mean values. (b) statistical comparisons were by Mann Whitney test, (e) statistical comparisons were by Kruskal–Wallis test with Dunn’s multiple comparison test. *p < 0.05, **p < 0.01, and NS, not significant.

Next, we wanted to determine whether the immunostimulatory potential of commensals identified in our *in vitro* macrophage screen reflected their effect on host immunity *in vivo*. We examined the ability of commensals from our panel identified as either high or low cytokine stimulators to regulate intestinal IL6 production as this cytokine plays a central role in the intestinal immune system during both homeostasis and disease [11,31]. To do this, we depleted the microbiota in mice using broad spectrum antibiotics. We found that IL6 production in the intestine is reduced after microbiota depletion demonstrating the importance of the microbiota for driving the homeostatic production of this cytokine in the intestine (Fig 1D). We then examined the ability of immunologically potent and immunologically weak stimulators of macrophages from all of the major intestinal phyla to regulate intestinal cytokine production after microbiota depletion. All of the potent macrophage activators from each phyla (Actinobacteria: *Bifidobacterium adolescentis*; Bacteroidetes: *Bacteroides caccae*; Firmicutes: *Enterococcus raffinosus*; Proteobacteria: *Acinetobacter radioresistens*) were able to rescue IL6 production after microbiota depletion (Fig 1D). All of the weak macrophage activators (Actinobacteria: *Bifidobacterium breve*; Bacteroidetes: *Bacteroides cellulosilyticus*; Firmicutes: *Eubacterium infirmum*; Proteobacteria: *Citrobacter koseri*) were significantly poorer at rescuing IL6 production than the robust stimulator from the same phylum (Fig 1E). This demonstrates that our macrophage screen accurately reflects the immunostimulatory activity of commensals in the intestine. Next, we wanted to test whether we could use this information on the immunostimulatory properties on individual commensals to predict the effect of more complex commensal communities on immunity *in vivo*. At the phylum level, we found that members of the Bacteroidetes tended to induce higher IL6 production, a product of a secondary response gene, than Firmicutes, but they had similar effects on TNF, the product of a primary response gene. This indicates that commensals from these phyla are likely to have different effects on programming our immune system and therefore led to two questions. Firstly, do multi-species communities of Bacteroidetes and Firmicutes have differential effects on these cytokines *in vivo*? And secondly, are the effects of these phyla generalizable to other primary and secondary response genes? To answer this, we inoculated microbiota-depleted mice (using antibiotic treatment) with consortium of either Bacteroidetes or Firmicutes and then measured the intestinal levels of the products of primary response genes (including TNF, CXCL1, CXCL2 and GM-CSF) and the products of secondary response genes (including IL6, IL12b and Lipocalin2) [29,30,33]. We found that our Bacteroidetes and Firmicutes consortia both induced primary gene product production (after both 4 and 24 hours) and there was no difference in the levels of primary gene products induced by these consortia (Fig 1E and 1F). By contrast, we found little induction of secondary genes by either the Bacteroidetes or the Firmicutes consortium at our early timepoint (Fig 1E), but at the later timepoint we found that Bacteroidetes induced significantly higher levels of secondary response genes than a consortium of Firmicutes at (Fig 1F), consistent with the slower induction kinetics of secondary response gene products. This pattern was consistent whether the commensals were alive or heat-killed (S4 Fig). To further corroborate these findings, we inoculated germ-free mice with either the Bacteroidetes or Firmicutes consortium. As with our microbiota depletion model using antibiotics, we found no difference in the ability of Bacteroidetes and Firmicutes to induce primary gene products but Bacteroidetes were better able to induce secondary gene products compared to Firmicutes (S5 Fig). Next, we wanted to understand whether macrophages were involved in the production of these cytokines downstream of commensals in the intestine. To do this we used liposome clodronate which specifically depletes macrophages prior to commensal inoculation. We found that after clodronate treatment there was reduced production of cytokines which were the product of both primary and secondary response genes (Fig 2G). By contrast, depletion of neutrophils had no effect on cytokine levels in the intestinal mucosa (S6 Fig). Collectively, these data begin to reveal taxonomic patterns in the effect of commensals on innate immunity, identify commensals with the potential to be major regulators of the innate immune system, and allow us to predict the immunological impact of complex commensal communities on the host based on their individual immunostimulatory properties. These taxonomic patterns in immune activation suggest that commensals within these two dominant phyla may engage different innate immune receptors and/or rely on different signaling pathways to exert their effects on the innate immune system.

### Bacteroidetes and Firmicutes exhibit different patterns in TLR activation and reliance for their effects on intestinal immunity

To examine potential differences in innate receptor stimulation, next we assessed the ability of our commensal panel to stimulate individual TLRs. TLRs are one of the principal classes of PRRs which mediate the effect of commensals on immunity[9]. We specifically focused on the activation of TLR2, TLR4 or TLR5 as these are the major TLRs which recognize bacteria and have established roles in mediating the effect of the microbiota on the immune system[11,12,14,16,22]. We assessed TLR activation by our commensal panel using in vitro HEK cell reporter cells expressing individual TLRs which couple receptor activation to the production of a secreted alkaline phosphatase which can them be measured spectrophotometrically[13]. Analogously to stimulation of macrophages, we found significant variation in commensal stimulation of TLRs (Fig 2A). Across the panel as a whole there was a broad distribution of TLR2 and TLR4 stimulatory potential (Fig 2A). By comparison, the distribution of TLR5 stimulation was more bimodal indicating that commensals were either potent TLR5 activators or provoked minimal activation of this receptor (Fig 2A). Comparison of mean SEAP production downstream of TLR stimulation elicited by individual species of the Bacteroidetes and Firmicutes revealed that Bacteroidetes tended to be more potent TLR4 activators than Firmicutes (Fig 2B). In comparison, there was no difference in TLR2 or TLR5 activation between these two major phyla (Fig 2B). Within the Firmicutes, commensals from the Clostridia class more potently activated both TLR4 and TLR2 compared to commensals from the Bacilli class (Fig 2B). As TLR4 is classically thought to be a receptor for LPS from Gram-negative organisms, rather than Gram-positives like Firmicutes, we did further controls to ensure that there was no contaminating LPS in our models. Firstly, to ensure that the overall immunostimulatory capacity of the Firmicutes that activate TLR4 was not due to LPS contamination, we stimulated macrophages with these organisms (*E*. *limosum*, *E*. *infirmum*, *C*. *histolyticum*, *P*. *anaerobius*, *C*. *orbiscindens*, *F*. *prausnitzii*, *L*.*reuteri*, *P*. *barengoltzii*, and *S*. *aureus*) with and without the LPS binding antibiotic polymyxin B present. We found that cytokine induction by these Firmicutes was unaffected by the presence of polymyxin B, whereas the stimulatory capacity of LPS was greatly diminished by polymyxin B (S7A Fig). Secondly, we stimulated our TLR4 reporter cells with these Firmicutes with and without polymyxin B present. Again, we found that TLR4 activation by these Firmicutes was unaffected by the presence of polymyxin B, whereas TLR4 activation by LPS was diminished by polymyxin B (S7B Fig). These data suggest that TLR4 activation by these Firmicutes was not due to contaminating LPS. Finally, to ensure that there was no LPS in the microbial growth media used to culture our Firmicutes which could have been carried over into our cell stimulation assays after washing the commensals, we stimulated macrophages with the media used to culture these Firmicutes with or without polymyxin B present. We found that the culture media elicited minimal cytokine production in macrophages and was unaffected by polymyxin B treatment indicating that there was no contaminating LPS in the growth media used to culture these Firmicutes (S7C Fig). Next, we wanted to determine whether we could identify the dominant TLR(s) responsible for mediating the immunostimulatory effects of commensals within our panel. To do this, we correlated macrophage cytokine production elicited by each commensal species with the degree to which they activate each TLR (Fig 2C). We found that for members of the Bacteroidia class their immunostimulatory capacity (measured by their ability to stimulate IL6 production in macrophages) correlated with how strongly they activated TLR4, but not TLR2 or TLR5 (Fig 2C). By contrast, the immunostimulatory capacity of Clostridia correlated with the robustness of both TLR2 and TLR4 activation (Fig 2C). The immunostimulatory effect of Bacilli did not correlate with the activation of any of the TLRs tested (Fig 2C). This led us to test whether members of these commensal classes within the Bacteroidetes and Firmicutes phyla rely on different TLRs to program host immunity *in vivo*. To test this, we blocked TLR signaling in microbiota-depleted mice using antibody neutralization prior to oral inoculation with a consortium of either Bacteroidia or Clostridia, and then determined the effects of these consortia on intestinal cytokine levels. We found that the stimulatory effect of Clostridia on intestinal IL6 production was only fully blocked after inhibition of both TLR2 and TLR4 indicating that the effect of this class of organisms is mediated by TLR2 or TLR4 activity (Fig 2D). By contrast we found that the regulation of intestinal IL6 production by Bacteroidia was blocked after neutralization of TLR4, but not TLR2 (Fig 2E). Together, these data reveal phylogenic patterns in TLR utilization required for different commensal taxa to exert their effects on the immune system.

### Canonical NF-κB signaling calibrates the immunostimulatory power of commensal communities

**Fig 3:**
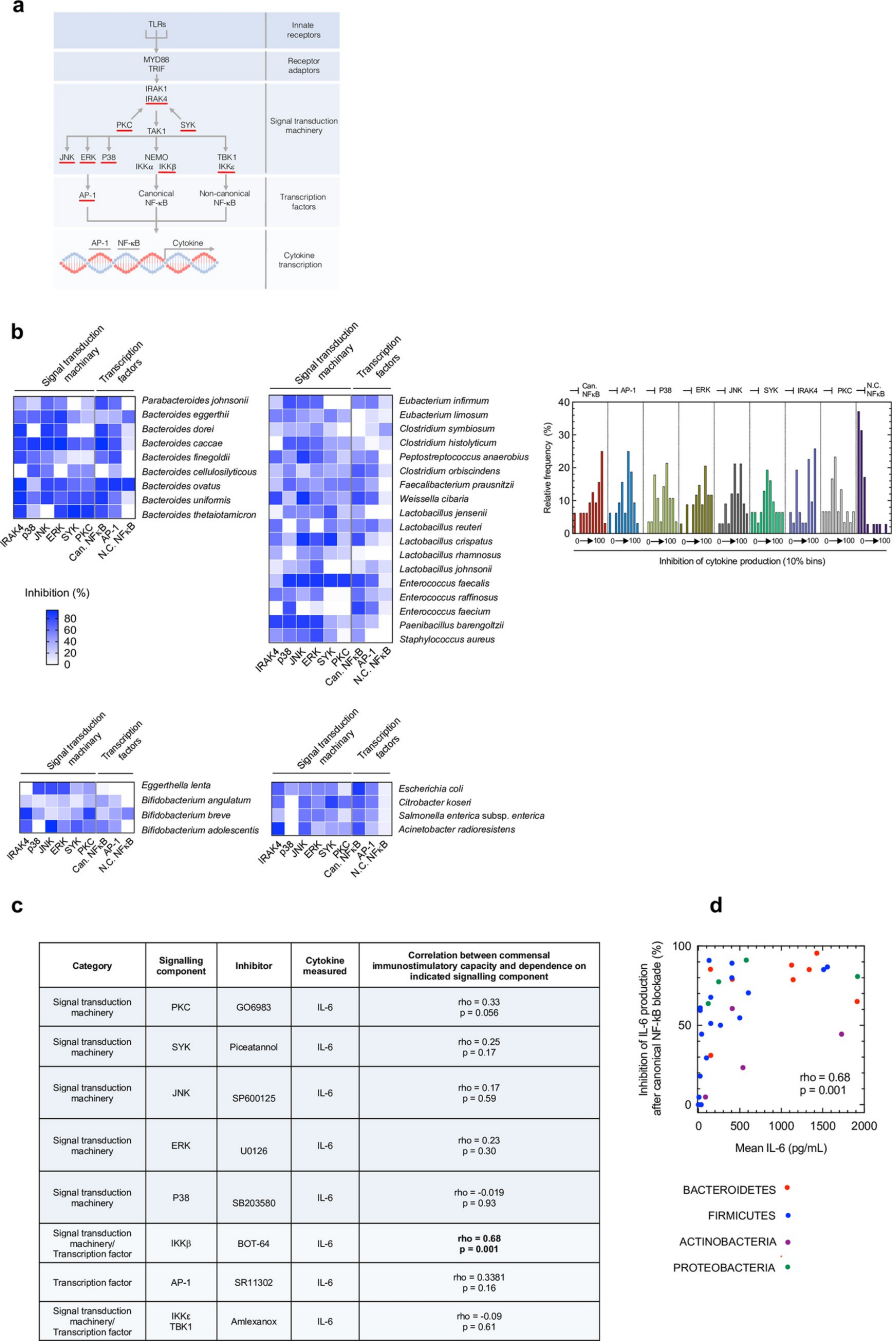
Canonical NF-κB is the ultimate determinant of the immunostimulatory power of commensals. (a) Schematic of important intracellular signaling components downstream of TLRs, components inhibited in this study underlined in red. (b) Heat map displaying percentage inhibition in IL6 production after pharmacological inhibition of IRAK4 (100 nM PF06650833), P38 (100 μM SB203580), JNK (10 μM SP600125), ERK (10 μM U0126), Syk (20 μM Piceatannol), PKC (10 nM GO6983), canonical NF-κB inhibitor IKKβ (10 μM BOT-64), non-canonical NF-κB inhibitor IKKε/TBK1 (100 μM Amlexanox) and AP-1 (50 μM SR11302). Data are relative to macrophages treated with vehicle control, with mean values for percentage inhibition for each species are shown (n = 4–10 biological repeats, at MOI of 1:10). Corresponding frequency distribution of cytokine inhibition for all commensals in our panel. (c) Correlation between overall immunostimulatory capacity of all commensals in our panel (measured by macrophage IL6 production) and their dependence on the indicated signaling component (measured by cognate reduction in cytokine production incurred after inhibition of each of the intracellular signaling components). (d) Correlation between overall immunostimulatory capacity of all commensals in our panel (measured by macrophage IL6 production) and their dependence on canonical NF-κB signaling (measured by cognate reduction in cytokine production incurred after inhibition of IKKβ). Correlations were determined by Spearman’s test with False Discovery Rate correction for multiple comparisons, adjusted p-values stated with p < 0.05 considered significant.

The transduction of microbial TLR stimulation into changes in cytokine production and immune cell behavior occurs via a limited number of intracellular signaling components[34]. These conserved intracellular pathways must integrate information from the concomitant activation of multiple TLRs by commensals, each activated at differing intensities and with different TLR ligand combinations, to determine changes in immune cell behavior and function downstream of commensal stimulation. How a limited number of signaling components achieve this information transfer to grade changes in immune cell behavior and cytokine production elicited by different commensals is poorly understood[35,36]. Next, therefore, we wanted to examine the intracellular signaling components required to transduce activation of innate receptors effect of each of our commensal species and identify any critical signaling components which determine the degree of immunostimulation elicited by commensals in our panel (Fig 3A).

We began by examining whether the IRAK4 signaling is required for the immunostimulatory effect of commensals in our panel. IRAK4 is a threonine/serine kinase that is essential for signaling downstream of MYD88 and hence is required for all TLR signaling except for TLR3[37]. Inhibiting IRAK4 therefore gives an indication of the role of TLRs in recognizing commensals in our panel. After pharmacological inhibition of IRAK4, we found that this inhibitor caused a significant reduction commensal-induced cytokine production for 27/35 of the commensals within our panel (Figs 3B and S8) highlighting the importance of TLRs for the immunostimulatory effect of commensals. The requirement for TLR signaling did, however, vary across our panel suggesting commensal species relied on TLRs to different extents to exert their immunostimulatory effects (Fig 3B). Next, we examined whether there were specific components of the intracellular signal transduction machinery required downstream of receptor and IRAK4 activation which control the immunostimulatory effect of commensals. To do this, we pharmacologically inhibited the MAP kinases (P38, ERK and JNK), protein kinase C and Syk. All of these signaling components are known to be important in meditating the effects of individual TLR activation on cytokine production and immune cell behavior[38–41]. We found that inhibition of the MAPKs had a pronounced effect on the immunostimulatory capacity of some commensals, for example *P*. *barengoltzii* and *E*. *limosum*, but had minimal effect on cytokine production driven by other commensal species, such as *B*. *angulatum* (Fig 3B). Inhibition of Syk and protein kinase C (α, β, γ, δ, ζ and μ isoforms) had a similar range of effects, with the immunostimulatory effect of some commensals highly dependent on Syk signaling, for example *B*. *thetaiotamicron* whereas the immunostimulatory effect of other species was independent of these pathways, for example *E*. *faecium* (Fig 3B). We then used this information to understand whether there were any broad trends in signaling component dependence across our commensal panel which could reveal critical parts of the intracellular signaling machinery which play a conserved role in dictating the degree of immunostimulation commensal communities exert. To do this, we correlated the immunostimulatory effect of each commensals in our panel with their dependence on components of the intracellular signal transduction machinery: MAPKs, Syk and PKC signaling. Specifically, we correlated mean macrophage IL6 production elicited by each commensal species (as a marker of commensal species immunostimulation) with the cognate reduction in IL6 production incurred after inhibition of each of the intracellular signaling components (as a marker for the dependence of each commensal species on that pathway for its immunostimulatory effect) (Fig 3C). We found no correlation between the level of cytokine production elicited by commensals in our panel and dependence on P38, JNK, ERK, Syk or PKC signaling (Fig 3C). Collectively, these data suggest that each commensal species has a distinct profile of intracellular signal components required to translate its pattern of innate receptor stimulation into changes in cytokine production. Furthermore, these data indicate that none of these components likely act as a conserved regulator dictating the level of immunostimulation commensal communities will elicit on host immunity. We then interrogated whether the transcription factors NF-κB or AP-1 acts as this critical control. We found that there was a significant positive correlation between the immunostimulatory capacity of commensals and their dependence on IKKβ of the canonical NF-κB signaling complex, but not AP-1 or non-canonical NF-κB (Fig 3C and 3D). To further substantiate these findings, we used siRNA to inhibit IKKβ of the canonical NF-κB signaling pathway (S9A Fig). As with pharmacological inhibition of IKKβ, we found that there was a significant positive correlation between the immunostimulatory capacity of commensals and their dependence on IKKβ (S9B Fig). This pattern was consistent within the Firmicute and Bacteroidetes phyla (S9B Fig). In contrast, and again in agreement with our data using pharmacological inhibition, we found there was no correlation between the immunostimulatory capacity of commensals and their dependence on JNK signaling (rho = 0.31, p = 0.07). Thus, signaling through the canonical NF-κB complex may act as a control hub to calibrate the immunological impact of commensal communities on the host.

### Rational design of commensal consortia to treat intestinal infections and inflammation

**Fig 4:**
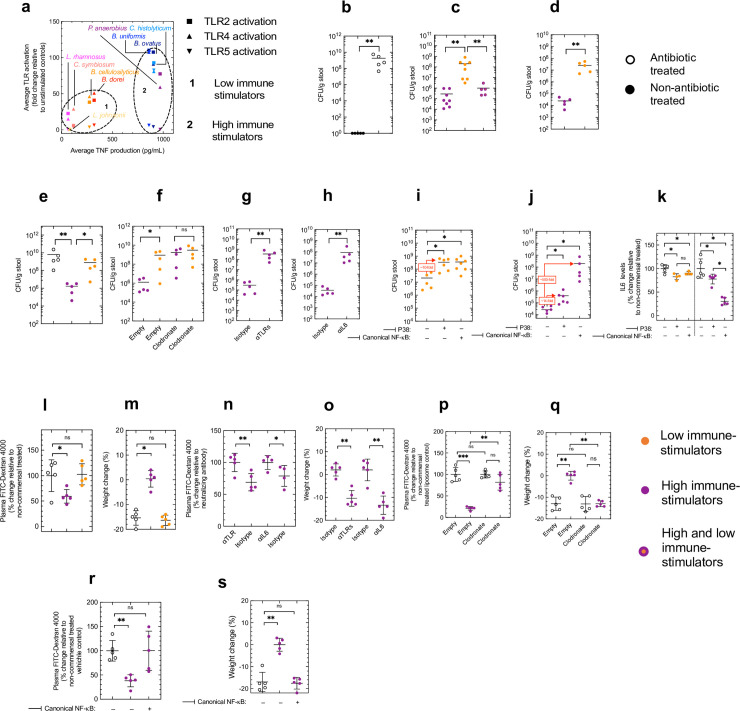
Rational design of commensal consortia to treat intestinal infection and intestinal inflammation. (a) Scatter plot of TLR activation (measured by TLR2-, TLR4- and TLR5-dependent SEAP activation) versus overall immunostimulatory capacity (measured by macrophage cytokine production) of indicated commensals from the Bacteroidetes and Firmicutes phyla. Commensals were grouped together into the “low immune-stimulators” (defined as <500 pg/mL TNF and <50-fold activation of all TLRs) and “high immune-stimulators” (defined as >500 pg/mL TNF and >50-fold activation of at least two TLRs). (b) VRE burden in the faeces of WT adult mice two days post-oral inoculation. Each point represents a single mouse and horizontal lines indicate median values. (c) VRE burden in the faeces of WT adult mice two days post-oral inoculation. Animals were orally inoculated with 1×10^8^ CFU of the “low immune-stimulating” commensal consortium, 1×10^8^ CFU of the “high immune-stimulating” commensal consortium, or 1×10^8^ CFU of the “low immune-stimulating” and “high immune-stimulating” commensal consortia combined, two days prior to VRE inoculation. (d) VRE burden in the faeces of WT adult mice four days post-oral inoculation. Animals were orally inoculated with 1×10^8^ CFU of the “low immune-stimulating” or “high immune-stimulating” commensal consortia two days post-VRE inoculation. (e) VRE burden in the faeces of adult SCID mice two days post-oral inoculation. Indicated animals were orally inoculated with 1×10^8^ CFU of the “low immune-stimulating” or “high immune-stimulating” commensal consortia two days prior to VRE inoculation. (f) VRE burden in the faeces of WT adult mice two days post-oral inoculation. Animals were orally inoculated with 1×10^8^ CFU of the “low immune-stimulating” or “high immune-stimulating” commensal consortia two days prior to VRE inoculation. Indicated mice were treated with clodronate liposomes or empty liposomes 2 days prior and concomitant with consortia inoculation. (g) VRE burden in the faeces of WT adult mice two days post-oral inoculation. Animals were orally inoculated with 1×10^8^ CFU of the “high immune-stimulating” commensal consortia two days prior to VRE inoculation. Concomitant with consortium inoculation mice were administered either isotype control or a cocktail of TLR neutralizing antibodies (αTLR2, αTLR4 and αTLR5, (100 μg/mouse)) via intraperitoneal injection. (h) VRE burden in the faeces of adult orally inoculated with the “high immune-stimulating” commensal consortia two days post-oral inoculation. Commensal consortia were administered two days prior to VRE inoculation. Concomitant with consortium inoculation mice were administered either isotype control or an IL6 neutralizing antibody via intraperitoneal injection (i,j) VRE burden in the faeces of WT adult mice orally inoculated with the “high immune-stimulating” commensal consortia two days post-oral inoculation with VRE. Commensal consortia were administered two days prior to VRE inoculation. For pharmacological inhibition of canonical NF-κB, mice were administered BOT-64 (30 mg/kg/dose) via intraperitoneal injection concomitant with consortia inoculation. For pharmacological inhibition of P38 mice were administered SB203580 (2 mg/kg/dose) via intraperitoneal injection concomitant with consortia inoculation. (k) Intestinal cytokine levels 24 hours post oral inoculation with 1×10^8^ CFU of indicated commensal consortium. Each point represents a single mouse and horizontal lines indicate median values. (l) Plasma FITC-dextran levels in mice with DSS-induced intestinal inflammation. For the induction of intestinal inflammation, antibiotic treated mice received 2% (w/v) DSS in drinking water for seven days. Indicated groups of mice were orally administered commensal consortia on day 2, day 4 and day 6 of DSS treatment. One day after cessation of DSS treatment, mice were orally administered 200 μL of FITC-dextran 4000 (80 mg/mL) and FITC-dextran 4000 levels in blood were measured after four hours. (m) Weight change in DSS-treated mice six days after start of DSS treatment. (n) Plasma FITC-dextran levels in mice with DSS-induced intestinal inflammation. For the induction of intestinal inflammation, antibiotic treated mice received 2% (w/v) DSS in drinking water for seven days. Indicated groups of mice were orally administered commensal consortia on day 2, day 4 and day 6 of DSS treatment. One day after cessation of DSS treatment, mice were orally administered 200 μL of FITC-dextran 4000 (80 mg/mL) and FITC-dextran 4000 levels in blood were measured after four hours. In indicated groups, concomitant with consortium inoculation mice were administered either isotype control or a cocktail of TLR neutralizing antibodies (αTLR2, αTLR4 and αTLR5, (100 μg/mouse)) or IL6 neutralizing antibody (100 μg/mouse), or isotype control via intraperitoneal injection. (o) Weight change in DSS-treated mice six days after start of DSS treatment. In indicated groups, concomitant with consortium inoculation mice were administered either isotype control or a cocktail if TLR neutralizing antibodies (αTLR2, αTLR4 and αTLR5, (100 μg/mouse)) or IL6 neutralizing antibody (100 μg/mouse), or isotype control via intraperitoneal injection. (p) Plasma FITC-dextran levels in mice with DSS-induced intestinal inflammation. For the induction of intestinal inflammation, antibiotic treated mice received 2% (w/v) DSS in drinking water for seven days. Indicated groups of mice were orally administered commensal consortia on day 2, day 4 and day 6 of DSS treatment. One day after cessation of DSS treatment, mice were orally administered 200 μL of FITC-dextran 4000 (80 mg/mL) and FITC-dextran 4000 levels in blood were measured after four hours. In indicated groups, concomitant with consortium inoculation, mice were treated with clodronate liposomes or empty liposomes concomitant with consortia inoculation. (q) Weight change in DSS-treated mice six days after start of DSS treatment. In indicated groups, concomitant with consortium inoculation, mice were treated with clodronate liposomes or empty liposomes concomitant with consortia inoculation. Each point represents a single mouse and horizontal lines indicate mean values. (r) Plasma FITC-dextran levels in mice with DSS-induced intestinal inflammation. For the induction of intestinal inflammation, antibiotic treated mice received 2% (w/v) DSS in drinking water for seven days. Indicated groups of mice were orally administered commensal consortia on day 2, day 4 and day 6 of DSS treatment. One day after cessation of DSS treatment, mice were orally administered 200 μL of FITC-dextran 4000 (80 mg/mL) and FITC-dextran 4000 levels in blood were measured after four hours. In indicated groups, for pharmacological inhibition of canonical NF-κB, mice were administered BOT-64 (30 mg/kg/dose) via intraperitoneal injection concomitant with consortia inoculation concomitant with consortium inoculation. (s) Weight change in DSS-treated mice six days after start of DSS treatment. In indicated groups, for pharmacological inhibition of canonical NF-κB, mice were administered BOT-64 (30 mg/kg/dose) via intraperitoneal injection concomitant with consortia inoculation concomitant with consortium inoculation. (b-h) Statistical comparisons were by Mann Whitney test, (i-s) statistical comparisons were by Kruskal–Wallis test with Dunn’s multiple comparison test. *p < 0.05, **p < 0.01, and NS, not significant.

Having determined the immunological fingerprint of commensals in our panel, next we wanted to use this information to rationally design communities of commensals to treat intestinal diseases by modulating specific components of the innate immune system and examine whether the effects of these commensal groups are determined by canonical NF-κB signaling. One of the most significant clinical problems caused by microbiota dysbiosis is increased susceptibility to enteric infection, a problem exacerbated by increasing antibiotic resistance[4,42]. Based on our commensal analysis, we assembled a cross-phylum consortium of commensals that demonstrated potent stimulation of macrophages (each species demonstrating induction of >500 pg/mL of TNF) and were robust activators of TLRs (each species demonstrating >50-fold activation of at least two TLRs) (Fig 4A). We called this our “high immune-stimulating” consortium. We chose to construct a cross-phylum consortium comprising both Firmicutes (from the Clostridia class), which rely on TLR2 and TLR4 for their effects on intestinal immunity (Fig 2D), and Bacteroidetes (from the Bacteroidia class), which rely on TLR4 only for their effects on intestinal immunity (Fig 2E), to ensure that the immunostimulatory effect of our consortium was not dependent on a single TLR pathway. As a comparison, we also assembled a consortium of commensals that were poor macrophage stimulators (each species demonstrating induction of <500 pg/mL of TNF) and weak TLR activators (each species demonstrating <50-fold activation of all TLRs), our “low immune-stimulating” consortium (Fig 4A). We then tested the ability of these commensals to enhance innate immune defenses against intestinal colonization by a major antibiotic resistant pathogen: vancomycin resistant Enterococci (VRE)[43]. In mice with an intact microbiota VRE was unable to establish intestinal colonization, by contrast after microbiota depletion VRE was able to establish a high level of intestinal colonization (Fig 4B), demonstrating that the microbiota is important for host defense against colonization by these organisms. To test the efficacy of the commensal consortia we had designed in the regulation of intestinal defenses, we orally inoculated microbiota-depleted mice with either the “high immune-stimulating” consortium or the “low immune-stimulating” consortium, prior to oral inoculation with VRE. As predicted from our analysis, the “high immune-stimulating” consortium provided significantly more protection against intestinal colonization by VRE than the “low immune-stimulating” group (Fig 4C). Furthermore, we found that the “low immune-stimulating” consortium did not inhibit the protective effect of the “high immune-stimulating” consortium against VRE (Fig 4C). The same pattern of protection if we established VRE colonization and then administered our consortia (Fig 4D). This pattern was maintained after heat-inactivation of our consortia (S10 Fig) supporting a role for PRR stimulation rather than metabolic products from our commensals regulating intestinal defenses. To ensure that there was equivalent engraftment of our “high immune-stimulating” and “low immune-stimulating”, we used phyla- and species-specific 16s rRNA gene qPCR, combined with bacterial culturing, of fecal samples to determine the *in vivo* levels of the commensals we administered to mice. Via 16s qPCR, we found similar levels of total bacteria, Firmicutes and Bacteroidetes between mice administered the “high immune-stimulating” and “low immune-stimulating” consortia (S11A Fig). There was similar relative abundance of each member of each consortium suggesting similar levels of commensal engraftment (11B and 11C and S11B Fig). To ensure that our consortia were viable within the intestine, we used selective fecal culturing. In comparison to feces from microbiota-depleted mice, and in agreement with our 16s qPCR data, the feces of mice administered our “low immune-stimulating” consortium had high levels of Lactobacilli, Bacteroidetes, and Clostridia (S10B and S10C Fig). Likewise, in comparison to feces from microbiota-depleted mice, and again in agreement with our 16s qPCR data, the feces of mice administered our “high immune-stimulating” consortium had high levels of Bacteroidetes, Clostridia and *P*. *anaerobius* (S11B and S11C Fig). Collectively these data indicate the differences in the effects of these consortia was not due to different intestinal engraftment. As our immunological profiling had been based on stimulation of innate immune cells, next we wanted to determine whether the effect of our commensal consortia *in vivo* was solely mediated by the innate immune system or whether the adaptive immune system was also involved. To do this, we orally inoculated our “high immune-stimulating” and “low immune-stimulating” consortium into microbiota-depleted SCID mice prior to oral infection with VRE. We found that our “high immune-stimulating” consortium was still more effective than our “low immune-stimulating” consortium even in the absence of adaptive immunity supporting the notion that our consortium is enhances innate defenses in the intestine (Fig 4E). We then wanted to examine the role of macrophages *in vivo*. To do this, we use clodronate liposomes to deplete intestinal macrophages[44]. Clodronate treatment abrogated the difference between the “high immune-stimulating” and “low immune-stimulating” consortia (Fig 4F), supporting the central role of macrophages downstream of consortia stimulation in enhancing intestinal antibacterial defences. We then tested whether this protection occurred through the stimulation of TLRs. Concomitant neutralization of TLR2, TLR4 and TLR5 abrogated the protective effect of our commensal consortia (Fig 4G), demonstrating that this consortium programs a protective immune barrier against a major antibiotic resistant pathogen through TLRs. Finally, given the central role of IL6 in intestinal immunity we tested whether this cytokine was involved in the protective effect of our “high immune-stimulating” consortium. After antibody neutralization, our “high immune-stimulating” consortium was less effective at promoting the clearance of VRE from the intestine (Fig 4H).

We then wanted to examine whether canonical NF-κB signaling calibrates the level of antibacterial defenses our commensal consortia elicit *in vivo*. To do this, microbiota-depleted mice were treated with an inhibitor of canonical NF-κB signaling concomitant with oral inoculation of our commensal consortia. As a comparison, we treated separate groups of microbiota-depleted mice with a P38 inhibitor concomitant with commensal consortia inoculation, which from our earlier analysis plays a role in translating the effect of commensals into changes in host immunity but would not be the critical determinant of the *degree* of immunostimulation our commensal consortia elicit. If our model is correct, then the dependence of the “high immune-stimulating” consortium on NF-κB signaling to promote intestinal defenses will be larger than the NF-κB-dependence of the “low immune-stimulating” consortium. By contrast, for MAPK signaling the magnitude of dependence on P38 signaling should not reflect the immunostimulatory power of our consortia. Consistent with our hypothesis, in mice given the “high immune-stimulating” consortium, we found that inhibition of NF-κB caused an approximately 500-fold increase in intestinal VRE levels (Fig 4I), whereas in animals given the “low immune-stimulating” consortium NF-κB inhibition caused only a 10-fold increase in intestinal VRE levels (Fig 4J). By contrast, in mice given the P38 inhibitor, VRE levels increased approximately 10-fold in both “high immune-stimulating” and “low immune-stimulating” consortia treated mice (Fig 4I–4J). The regulation of intestinal cytokine levels by are rationally designed commensal consortia displayed a similar pattern of dependence (Fig 4K). Collectively, our data show that whilst a variety of intracellular signaling pathways are involved in translating commensal stimulation into changes in immune behavior it is ultimately NF-κB through IKKβ which acts as a critical control hub to calibrate the strength of the effect of commensal communities on immunity.

Stimulation of TLR signaling by the microbiota not only enhances intestinal antibacterial defenses but it is also required to restore intestinal homeostasis during pathological inflammation[11]. We therefore reasoned that the ability of the commensal consortia that we had designed to effectively engage TLR signaling would not only enhance antibacterial defenses in the intestine but could also be used to combat intestinal damage due to pathological inflammation. To test this, microbiota-depleted mice were treated with dextran sulfate sodium (DSS) to induce intestinal inflammation and then orally administered FITC-dextran 4000 to assess the integrity of the mucosal barrier. Consistent with previous work, we found FITC-dextran 4000 in the circulation of DSS-treated mice indicating that inflammation disrupted mucosal barrier function, and we found that circulating levels of FITC-dextran 4000 decreased after administration of the “high immune-stimulating” consortium but not the “low immune-stimulating” consortium (Fig 4L). Similarly, we found that weight loss induced by DSS treatment was reduced after administration of the “high immune-stimulating” but not the “low immune-stimulating” consortium (Fig 4M). Next, we wanted to examine the mechanistic basis for how our “high immune-stimulating” consortium restores intestinal barrier function. First, we wanted to examine whether protection occurred through the stimulation of TLRs. Using antibody neutralization, we found that blockade of TLR signaling eliminated the protective effect of our “high immune-stimulating” consortium on intestinal homeostasis (Fig 4N–$O). We then wanted to understand what signals are translating TLR stimulation into intestinal homeostasis. As IL6 plays a fundamental role in intestinal homeostasis we neutralized this pathway to test its role in mediating the effect of our “high immune-stimulating” consortium on intestinal homeostasis. We found that the beneficial effects of our consortium were lost after inhibition of IL6 (Fig 4N–4O) demonstrating that this pathway plays an integral role in mucosal homeostasis downstream of our “high immune-stimulating” consortium. To examine the role of macrophages, we depleted these cells using clodronate treatment prior to commensal consortia inoculation. After clodronate treatment, our “high immune-stimulating” consortium could no longer restore mucosal barrier function and prevent weight loss, indicating that macrophages are required for the restorative effect of the “high immune-stimulating” consortium (Fig 4P–4Q). Finally, we wanted to test whether analogously to the protective effect of our “high immune-stimulating” consortium on intestinal defenses, we wanted to know whether canonical NF-κB signaling played a role in restoring mucosal barrier function during intestinal inflammation. In mice given the “high immune-stimulating” consortium, we found that inhibition of canonical NF-κB prevented the “high immune-stimulating” consortium restoring mucosal barrier function and protecting against weight loss (Fig 4R–4S). Collectively, our study shows that we can rationally design commensal groups based on their immunological profiles to combat infectious and inflammatory diseases associated with microbiota dysbiosis through manipulation of intestinal innate immunity.

## Discussion

Programming of immune development and function depends upon microbial signals from diverse taxonomic groups of commensal bacteria[1,2,5,9,10]. The immunological potential of different members of the microbiota has been poorly defined meaning that our understanding of who the critical microbial regulators of immunity are during health and disease is severely limited. In this study, we have provided detailed insight into the immunostimulatory activity of commensal bacteria from the major taxa in the human intestinal microbiota and used this information to rationally construct commensal consortia to modulate intestinal immunity. Through this immunological design approach, we developed commensal consortia which broadly fortify innate immune defenses against antibiotic resistant pathogens and combat pathological intestinal inflammation. We identified the innate receptors, the intracellular signaling components, cytokines, and cell type which form the signaling axis that assimilate the microbial cues from our commensal consortia and translate this into enhanced antibacterial defenses and restore mucosal barrier function. Within this pathway, we find that canonical NF-κB signaling acts as the ultimate controller of the effect of commensals on the immune system by calibrating the degree of innate immune stimulation and antibacterial immunity elicited by our commensal consortia.

Predicting how changes in the taxonomic composition of the microbiota impacts host physiology remains a significant challenge limiting rationale microbiota reengineering to treat disease [5,45]. This is because, currently, how a reduction or increase in abundance of a certain taxa subsequently affects immunity is unclear because we do not know the immunological impact different commensals have on the host. The approach we use here, characterizing the immunological properties of commensals on the innate immune system, begins to address this challenge. One of the most striking taxonomic patterns in immunostimulation we identified was that, for the Bacteroidetes and Firmicutes that we tested, Bacteroidetes tended to be more effective at stimulating the production of secondary response genes than Firmicutes. There was no difference in their effect on primary response genes. Based on this, we hypothesized that a community of Bacteroidetes would stimulate the production of secondary response genes in the intestine to higher levels than a community of Firmicutes. This hypothesis was correct and likely drives differences in the programming of intestinal immunity by members of these two dominant phyla. For example, the higher levels of Lipocalin2 induced by a consortium of Bacteroidetes, compared to a consortium Firmicutes suggests that Bacteroidetes could be more robust stimulators of basal antibacterial defenses than Firmicutes. Our data could therefore help to explain why Bacteroidetes protect against intestinal colonization by *Klebsiella pneumoniae* better than Firmicutes[44]. Conversely, a domination of Bacteroidetes in the intestine could lead to excessive immune stimulation, as has been noted in some models of intestinal inflammation[46,47]. This could provide a immunological rationale for why maintaining the ratio of Firmicutes to Bacteroidetes within an ideal range is critical for host health[48,49].

What causes these taxonomic patterns in primary and secondary response gene induction likely reflect the taxonomic differences in TLR activation we uncovered. Whilst the effect of TLR stimulation by the whole microbiota on immunity is well defined how different taxonomic groups within the microbiota engage these receptors and whether different commensal taxa rely on different TLRs to exert their immunoregulatory effects has remained unclear. By analyzing a broad range of commensals, we could link the immunostimulatory capacity of different commensal taxa to specific TLR signaling pathways. At the species level, for example, microbiota engineering to increase the absolute colonization levels of *Clostridium symbiosum* would increase TLR2 signaling and, likewise, increased *Bacteroides uniformis* would increase TLR4 activity. By contrast, replacement of these species by *Clostridium histolyticum* and *Bacteroides cellulosyticous* respectively, would dampen TLR2 and TLR4 activity. Our results also indicate how changes at a higher taxonomic level, for example at the class or phylum level, could impact immunity via TLRs. For example, an increase in Bacteroidetes levels, compared to Firmicutes, would likely lead to an increase in TLR4 stimulation. We found that the immunostimulatory effect of commensals from the Bacteroidia class correlated with the degree to which they activated TLR4, for the Clostridia class immunostimulatory capacity reflected the robustness of TLR2 and TLR4 activity, whereas there was no clear pattern for Bacilli. For Bacteroidia this does not simply reflect an absence of TLR2 agonists as many species of Bacteroidia were able to activate TLR2 and activation of TLR2 by Bacteroidetes capsular polysaccharide has been documented[16]. It suggests, however, that their overall immunostimulatory capacity was dominated by their ability to activate TLR4. This is supported by our experimental data analyzing the pathways required for the regulation of immunity *in vivo*. We demonstrated that intestinal cytokine production driven by Bacteroidia was dependent on TLR4 alone, whilst the effect of Clostridia can be mediated by signaling via both TLR2 and TLR4. What drives these differences is currently unclear but likely mirrors the cell wall structure of commensals within these taxa. For the Bacteroidia the requirement of TLR4 alone is likely due to the dominance of the LPS ubiquitous within this class of bacteria[50–52]. These organisms may produce other immunostimulatory molecules that activate other PRRs but our work suggests that it’s the degree to which they activate TLR4 which really drives their immunostimulatory effect. The immunostimulatory capacity of commensal Clostridia relies on TLR2 and TLR4. Ligands for TLR2 are produced by pathogenic and non-pathogenic Clostridia[53–55], but how these, and other TLR4 activating Firmicute species which do not produce LPS activate TLR4 is more mysterious. A number of non-LPS TLR4 agonists have been identified however, it is unclear whether these are true ligands or artifacts of *in vitro* model systems[56,57]. Our data demonstrating that the effects of Clostridia *in vivo* requires TLR4 signaling supports the fact that these non-LPS producing organisms are true activators of TLR4. Furthermore, in our *in vitro* models, we used a number of approaches to eliminate the possibility of LPS contamination. While classically regarded as a receptor for LPS, there is a substantial body of literature on non-LPS activators of TLR4. These molecules are predominantly either other cell wall polymers or proteins. One of the original reports identifying the ligands which activate TLRs demonstrated that *S*. *aureus* lipoteichoic acid activates TLR4[58]. More recently peptide toxins produced by *S*. *aureus*, phenol soluble modulins, have also been shown to activate TLR4[59]. Another bacterial toxin, Anthrolysin O from *Bacillus anthracis* can also activate TLR4[60]. Similarly, fungi, because of their complex cell walls, are also a rich source of non-classical TLR4 activators. For example, *O*-linked mannosyl moieties in the cell wall of *Candida albicans* activate TLR4[61], and the capsular polysaccharide of *Cryptococcus neoformans*, glucuronoxylomannan, also activates TLR4[62]. For pathogenic Clostridia their surface layer has been demonstrated to activate TLR4[53], whether a similar phenomenon occurs with the surface layer of commensal Clostridia is unclear, but if it did it could account for the activation of TLR4 by members of this commensal class. How structurally unrelated molecules can activate a single TLR has remains unclear, but recent structural biology studies developing synthetic TLR4 agonists has shown that molecules with no structural similarity to LPS are able to bind and activate TLR4[63]. These differences in TLR activation patterns between members of the Bacteroidetes and Firmicutes could therefore underlie the differences between these phyla on primary and secondary response genes. This is because it has been demonstrated that ligation of TLR4 by LPS stimulates the production of a number of secondary response genes (including IL6 and IL12b) more potently than stimulation of TLR2 by a synthetic ligand[33]. This is thought to be due to the ability of TLR4 to signal through MYD88 and TRIF, whereas TLR2 signals through MYD88 alone. The more potent activation of TLR4 by Bacteroidetes could therefore underlie this difference in primary and secondary response gene induction.

Downstream of TLR activation changes in immune cell behavior and cytokine production are relayed by a limited number of intracellular signal transduction components, such as the MAPKs and NF-κB. How these components transduce the stimulation of a single TLR into changes in immune behavior and cytokine production is well characterized[29,30,33,34]. By contrast, how these intracellular components relay more complex patterns of TLR activation, as will occur downstream of commensal stimulation with multiple TLRs activated at different strengths and in different combinations, is unclear[35,36,64]. We found different selectivity in the pathways each commensal species relies upon to stimulate cytokine production with no clear patterns in the reliance on MAPKs, PKC or Syk. This idiosyncratic pattern of pathway activation for each species means that the stimulatory effect of a whole commensal community is unlikely to be reliant on a single one of these intracellular components. Our analysis did, however, identify one critical signaling component downstream of receptor activation: IKKβ of the canonical NF-κB signaling complex. We found that for both Bacteroidetes and Firmicutes, the more immunostimulatory the commensal species, the more it relied on canonical NF-κB. Thus, NF-κB is a bottleneck in the transfer of information from upstream receptor activation to downstream changes in immunity. Consequently, for a given commensal community its stimulatory effect on immunity will be transmitted through the activity of a variety of intracellular signaling components with all of this stimulatory information then funneled through IKKβ which will be the ultimate determinant of how powerful the effect of the commensal community will be on the host. This role of NF-κB has a number of implications. Firstly, it suggests that a single protein–IKKβ–has to assimilate all upstream signaling information and encode it in downstream changes in gene transcription. This is a significant information transfer bottleneck that, if disrupted, will be likely to severely disturb the effect of commensals on innate immunity. Secondly, it supports the notion that in conditions of dysregulated intestinal immunity with an overt inflammatory component, blockade of canonical NF-κB could be the most effective pathway to target to reduce disease severity.

Macrophages were central to the effect of our commensal consortia on intestinal immunity in line with their critical role in the mucosa during homeostasis and disease. During homeostasis intestinal macrophages develop reduced responses to TLR stimulation[27,65,66], however, during intestinal inflammation and infection this anergy is lost and they secrete more cytokines in response to microbial stimulation[67]. This strongly supports the utility of our commensal consortia-based approach in disease settings when intestinal macrophages will be most responsive to signals from our commensal consortia. This does not mean, however, that our findings may not be relevant to understanding the effect of commensals during homeostasis. While cytokine production and response to TLR stimulation is reduced in intestinal macrophages it is not eliminated[66,68], possibly reflecting that intestinal macrophages are constantly replenished by circulating monocytes and these cells have to be conditioned upon arrival in the intestine to control their inflammatory potential[65]. Intestinal macrophages isolated from mice in the absence of infection or inflammation do produce low levels of cytokines classically described as proinflammatory, such as TNF and IL6, in response to TLR stimulation [66,69] but, crucially, they also produce IL-10 to temper their activity[66]. This balance is likely critical to maintain the “physiological inflammation” that it required for intestinal homeostasis[70]. The balance between these two seemingly opposing forces is most strikingly demonstrated by studies which show intestinal mucosa dysfunction not only in mice deficient in IL-10 [71] but also evident in animals deficient in IL6 [72].

We demonstrated the value of delineating the TLR stimulating potential of commensals by designing consortia that enhance defenses against antibiotic resistant pathogens and restore mucosal barrier function during pathological inflammation. This is significant for a number of reasons. Firstly, it demonstrates that our data assessing the immunostimulatory capacity of commensals generated using macrophages and TLR reporter cells provides an accurate prediction of the effect of commensals on protecting against intestinal infection *in vivo*. We found that heat-killed and viable commensals elicited a similar response *in vivo* too. These findings suggest that the immunological impact of the commensals in our panel is dominated by their ability to activate host factors which recognize conserved structural components (i.e. PRRs) rather than the effect of metabolic products produced by commensals. Secondly, we found that the immunostimulatory capacity of individual commensals accurately predicts the behavior of more complex multiple species communities on immunity *in vivo*. There are likely to be synergistic or antagonistic interactions between commensals that mean that understanding the immunological properties of multiple commensal species individually might not reflect their collective effect on immunity *in vivo*. Our data show that for the regulation of intestinal antibacterial defenses by commensals there is a direct relationship between the individual immunological impact of commensals and their impact as a community. Thus, if we know the immunological properties of a given commensal species we can predict how an increase or decrease in its abundance will impact intestinal immunity. Thirdly, our rationally designed consortia of robust TLR activators was protective against VRE. This is a significant threat to human health because of its resistance to currently used antibiotics[43]. Thus, new therapeutic approaches to treat this and other infections by similar pathogens is required. While microbiota-based approaches to achieve this have been widely proposed they currently suffer from a number of drawbacks. The first approach is to use whole fecal microbiota transplantation. For the currently tested pathogens, such as *C*. *diffcile*, this has the potential advantage of being highly efficacious[73]. However, the requirement for fecal screening to eliminate the transplantation of potentially deleterious microbes which can cause infections and negatively impact host metabolism reduces its utility[73]. A more refined method to circumvent this problem, is to use defined commensal communities to protect against infection[8,23,42,74]. This approach relies on the identification of commensals which are protective against infection. This has been the major bottleneck in this approach, with only a few protective commensals identified and for most of the identified commensals their protective effects have been identified retrospectively[13,75–77]. That is, by first identifying that the complete microbiota protects against a specific pathogen, and then by painstaking deduction of which commensals are the dominant protective species. This retrospective approach has dominated because it has remained extremely difficult to predict how different commensals will impact host defenses. We believe that our prospective approach of delineating the immunological properties of individual commensals, which can then be used to inform their deployment *in vivo*, addresses this problem and expediates the process of developing protective commensal communities by allowing rational construction of consortia based on the known immunological properties of each commensal species. Similarly, microbiota manipulation has been proposed as a way of combating chronic inflammatory conditions within the intestine, such as Crohn’s and ulcerative colitis[78]. Current approaches to harness the power of the microbiota for this remain largely trial-and-error, with little theoretical underpinning to predict why certain commensals or probiotics might be suitable candidates to treat these conditions. Again, our work tackles this problem by using immunological information to identify commensals which can treat intestinal inflammation. However, further understanding of the basic pathobiology of these chronic inflammatory conditions is required as the role of TLR signaling is often specific to the stage in disease with opposing effects[79]. Therefore, depending on the disease stage it might be more suitable to try to reengineer the microbiota with a low TLR stimulating group of commensals compared to a robust group of TLR stimulating organisms. The beauty of the approach outlined in this work is that it allows for the rational construction of commensal consortia for either scenario. Thus, our work provides a unique resource to understand the microbiota and disease. Through our elaboration of the immunological properties of individual human intestinal commensals it allows the selection of individual commensals of the required immunological fingerprint to treat disease making rational microbiota reengineering a realistic therapeutic proposition.

## Materials and methods

### Ethics statement

The use of mice was performed under the authority of the UK Home Office outlined in the Animals [Scientific Procedures] Act 1986 after ethical review by Imperial College London Animal Welfare and Ethical Review Body (PPL 70/7969 and PF93C158E).

### Bacterial strains

Bacteria used in this study and their growth conditions are outlined in S1 Table.

### Macrophage culture, stimulation and inhibitor treatment

The murine macrophage cell line J774A.1 was cultured in DMEM supplemented with 10% v/v FBS, penicillin (100 units/mL) and streptomycin (100 μg/mL) and maintained at 37°C and 5% (v/v) CO_2_. The human monocyte cell line was maintained in RPMI 1640 with 1×GlutaMAX, 10% FBS, penicillin (100 units/mL) and streptomycin (100 μg/mL), 1 mM sodium pyruvate and 10 mM HEPES. Cells were passaged via gentle scraping. THP-1 monocytes were differentiated to macrophages by treatment with PMA (100 ng/mL) for 48 hours. For bone marrow derived macrophages, C57BL/6 mice were humanly sacrificed using a Schedule 1 method. All fur and skin were removed from the legs, the muscle cut away until the bone was completely exposed. To remove the leg, a cut was made above the leg joint without removing the top of the femur. The bones were cleaned of any remaining tissue and placed in a petri dish containing PBS. Bones were transferred into a petri dish containing 70% ethanol for 1 minute then returned to the PBS. To remove the femur, the hip joint was cut off whilst holding the bone with sterile tweezers then similarly the knee joint was cut. To remove the tibia, the knee joint was removed, then the tibia was cut just below where the red bone marrow ends. The bone marrow was flushed into a 50 mL falcon tube by inserting a 26-gauge needle attached to a 20 mL syringe filled with PBS. The marrow was dispersed by aspirating it into an empty 20 mL syringe through a 19-gauge needle twice, then the cells were centrifuged at 1,500 rpm for 5 minutes at 4°C. Cells were then re-suspended in 15 mL of R10 medium containing 15% L-cell conditioned media (LCM). The cell suspension was used to seed flasks containing 20 mL R10 media + 15% LCM which were incubated at 37°C and 5% CO_2_. For macrophage stimulation, J774A.1 macrophages or BMDMs were removed from culture flasks by gentle scrapping and seeded at a density of 2.5×10^4^ cells/well in a 96 well plate in 100 μL of growth media and incubated overnight at 37°C and 5% CO_2_. Supernatant was removed and 100 μL of fresh growth media added. Macrophages were challenged with heat-killed bacteria at an MOI of 1:10 and incubated overnight in the same conditions. The supernatant was then removed, and cytokine production measured by ELISA. 100 μL of fresh growth media was added to the cells with inhibitors, as described in figure legends. The cells were incubated at 37°C and 5% CO_2_ for the pre-incubation time stated then heat-killed (65°C for 30 mins) bacteria were added at MOIs of 1:10. Cells were incubated overnight at 37°C and 5% CO_2_ then the supernatant was removed, and the cytokine production measured by ELISA. For treatment with inhibitors, macrophages were incubated with indicated inhibitors for 1 hour prior to commensal stimulation. Inhibitors used were: U0126 (ERK 1/2 inhibitor (Cell Signaling Technologies)); SP600125 (JNK inhibitor (Fluorochem)); SB203580 (P38 inhibitor (InvivoGen); PF 06650833 (IRAK4 inhibitor (TOCRIS); BOT-64 (IKK-β inhibitor (Sigma)); GO 6983 (PKC isoforms α, β, γ, δ, ζ and μ inhibitor (Abcam)); Piceatannol (Syk inhibitor (Sigma)); and SR11302 (AP-1 inhibitor (TOCRIS)) at indicated concentrations. All inhibitors were tested to ensure they were not toxic at concentrations used. Polymyxin B (Tokyo Chemical Industries) was used at indicated concentrations. siRNA oligonucleotides against IKK-β (Ambion), JNK1 and JNK2 (Ambion), and cognate control siRNAs were used to transfect J774A.1 cells using Lipofectamine RNAiMAX transfection reagent according to the manufacturer's instructions. Cells were used 24 hr post-transfection. Cell death was determined by measuring Lactate Dehydrogenase release using the CyQUANT LDH Cytotoxicity Assay kit according to the manufacturer’s instructions (Invitrogen).

### HEK cell culture and stimulation

HEK Blue cells expressing human TLR2 (catalog number hkb-htlr2), TLR4 (catalog number hkb-htlr4) and TLR5 (catalog number hkb-htlr5) (InvivoGen), and control HEK Blue Null cells (catalog number hkb-null) (InvivoGen) were cultured at 37°C in 5% CO_2_ in Dulbecco’s modified Eagle's media supplemented with 10% (v/v) fetal bovine serum (Invitrogen), penicillin (100 units/mL), streptomycin (100 mg/mL), blastocidin (30 mg/mL), and zeocin (100 mg/mL). Cells were then seeded at 2.5×10^4^ cells/well in a 96 well plate in growth media and allowed to adhere overnight. Supernatant was removed and 100 μL of fresh growth media was added. HEK-blue cells were challenged with heat-killed bacteria at a multiplicity of infection of 1:10. HEK-blue NULL2 cells being utilized as a negative control for receptor stimulation. Cells were then incubated at 37°C and 5% CO_2_ for 24 hours. 50 μL of supernatant was removed and heat-inactivated at 65°C for 5 minutes then added to 150 μL of Quanti-Blue detection media (InvivoGen). Alkaline phosphatase activity was measured on a spectrophotometer at 620 nm (OD620).

### Cytokine quantification

Cytokines levels were determined using ELISA kits for TNF, IL6, GM-CSF (all Biolegend), CXCL2, CXCL1, IL-12B, LCN2 (all Thermo Fisher Scientific) according to the manufacturer’s instructions.

### Mice, microbiota and cell depletion

Wild-type C57BL/6 mice were purchased from Envigo (UK) and CB17 SCID mice were from Charles River (UK), germ-free Swiss Webster mice were purchased from Taconic. All adult mice were female and normally between 6 − 14 weeks old, except germ-free mice which were male and female. Adult mice were housed no more than five per cage with Aspen chip 2 bedding with a 12 hour light and 12 hour dark cycle at 20°C − 22°C. Mice were randomly assigned to experimental groups, water was provided ad libitum and mice were fed RM1 (Special Diet Services). To deplete the microbiota, mice were given broad-spectrum antibiotics (metronidazole 1 g/L, neomycin sulfate 1 g/L, ampicillin 1 g/L, and vancomycin 0.5 g/L) in drinking water for 7–10 days[13,80]. Macrophages were depleted using clodronate-containing liposomes (FormuMax Scientific) as described previously [13,44] confirmation of depletion shown in (S12 Fig). Neutrophils were depleted using the anti-mouse Ly-6G antibody (1A8) (Biolegend) and confirmed by measuring intestinal MPO levels (S13 Fig).

### Models of bacterial colonization in the intestine

To establish intestinal colonization by VRE unanaesthetized mice were orally inoculated with approximately 5×10^5^ CFU of VRE in 200 μL of PBS. For consortia inoculation mice were orally inoculated with 5×10^8^ CFU of the indicated commensal consortia two days prior to VRE inoculation. Macrophages were depleted using clodronate liposomes as described in[44]. Engraftment of consortia was measured by qPCR of 16s rRNA gene as described in[13,44] using the following primers: 16s forward 5’-ACTCCTACGGGAGGCAGCAGT-3’; 16s reverse 5’-ATTACCGCGGCTGCTGGC-3’; Firmicutes forward 5’-GGAGYATGTGGTTTAATTCGAAGCA-3’; Firmicutes reverse 5’-AGCTGACGACAACCATGCAC-3’; Bacteroidetes forward 5’-GGARCATGTGGTTTAATTCGATGAT-3’; Bacteroidetes reverse 5’-AGCTGACGACAACCATGCAG-3’. *B*. *dorei* forward 5’-GGAAACGGTTCAGCTAGCAATA-3’; *B*. *dorei* reverse 5’-AGTCTTGTCAGAGTCCTCAGCATC-3’. *B*. *ovatus* forward 5’-CCGGATAGCATACGAATATC-3’; *B*. *ovatus* reverse 5’-CGTAGGAGTTTGGACCGTGT-3’. *L*. *johnsonii* forward 5’-GATTTTAGTGCTTGCACTAAA-3’; *L*. *johnsonii* reverse 5’-ATTACCTTACCAACTAG-3’. *C*. *symbiosum* forward 5’-GGGAACTCTTGGGAGACTGC-3’; *C*. *symbiosum* reverse 5’-TGACTCCCATGGTGTGACG-3’. *L*. *rhamanosus* forward 5’-GTGCTTGCATCTTGATTTAATTTT-3’*; L*. *rhamanosus* reverse 5’-TGCGGTTCTTGGATTTATGCG-3’. *C*. *histolyticum* forward 5’-AGAAGCAAGACCGTGAGGTG-3’; *C*. *histolyticum* reverse 5’-CGGACTTCGGGTGTTACCAA-3’. *P*. *anaerobius* forward 5’-CGATGAGTACTAGGTGTCGGG-3’; *P*. *anaerobius* reverse 5’-AGCCCCGAAGGGAAGGTGTG-3’. *B*. *ovatus* forward 5’-TCTTCCGCATGGTAGAACTATTA-3’; *B*. *ovatus* reverse 5’-ACCGTGTCTCAGTTCCAATGTG-3’. *B*. *cellulosilyticus* forward 5’-AGGATGACTGCCCTATGGGT-3’; *B*. *cellulosilyticus* reverse 5’-CTGCACTCAAGACGACCAGT-3’. In mice administered our “high immune-stimulating” and “low immune-stimulating” consortia fecal levels of viable Lactobacilli were determined by culture on Lactobacillus-selective MRS media, Bacteroides by culture on Bacteroides-selective Bacteroides Bile Esculin (BBE) media, and Clostridia by culture on Modified Reinforced Clostridial media (MRCA) (with Bacteroidetes level subtracted as MRCA can support growth of Bacteroidetes as well as Clostridia). *P*. *anaerobius* levels were determined by culture on TSA media +/- sodium polyanethol sulfonate at 0.05%. Sensitivity to sodium polyanethol sulfonate is used to identify *P*. *anaerobius*[81] and the other members of the consortium *P*. *anaerobius* is in are insensitive to it.

### Signaling pathway neutralization *in vivo*

For antibody neutralization, mice were administered via the intraperitoneal injection αTLR2 (InvivoGen), αTLR4 (eBiosciences), αTLR5 (InvivoGen), or αIL6 (eBiosciences) neutralizing antibodies (100 μg/mouse) or pertinent isotype control, as indicated, three days prior to, and concomitant with, consortia inoculation. For pharmacological inhibition of canonical NF-κB, mice were administered BOT-64 (30 mg/kg/dose) via intraperitoneal injection concomitant with consortia inoculation. For pharmacological inhibition of P38, mice were administered SB203580 (2 mg/kg/dose) via intraperitoneal injection concomitant with consortia inoculation.

### Intestinal inflammation and permeability assay

For the induction of intestinal inflammation, antibiotic treated mice received 2% (w/v) DSS in drinking water for 7 days[11]. Indicated groups of mice were orally administered commensal consortia on day 2, day 4 and day 6 of DSS treatment. Intestinal barrier function was examined by measuring FITC-dextran 4000 levels in blood. Mice were orally administered 200 mL of FITC-dextran 4000 (80 mg/mL) one day after cessation of DSS treatment and fluorescence intensity in the blood was measured four hours after FITC-dextran inoculation (excitation: 495 nm and emission:519 nm).

### Phylogenetic tree construction

The phylogenetic tree was constructed using the NCBI’s Taxonomy database to create a common tree which was then visualized using FigTree v1.4.4.

### Statistical analysis

Statistical comparisons were performed using GraphPad Prism software. To compare differences between two groups, the Student’s t-test or the Mann–Whitney test were used. For all other comparisons a one-way analysis of variance (with post-hoc Sidak’s test or Dunnett’s test), a Kruskal–Wallis test with Dunn’s multiple comparison test, were used as appropriate. Correlations were determined using either the Pearson’s or the Spearman’s correlation test with FDR used to correct for multiple comparisons[82]. P-values <0.05 were considered significant.

## Supporting information

S1 Fig Correlation between cytokine production induced by commensals from J774A.1 macrophages and bone marrow-derived macrophages. TNF production at MOI of 1:10 induced by *B*.*cellulosilyticus*, *B*. *dorei*, *B*. *caccae*, *W*. *cibaria*, *E*. *faecium*, *L*. *rhamnosus*, *C*. *symbiosum*, *C*. *orbiscindens*, *L*. *reuteri*, *L*. *johnsonii*, *L*. *crispatus*, *L*. *jensenii*, *A*. *radioresistens* and *C*. *koseri*. Correlation was determined using the Pearson’s test.
(TIF)

S2 Fig Correlation between cytokine production induced by commensals from J774A.1 macrophages and differentiated THP-1 macrophages. TNF production at MOI of 1:10 induced by *B*.*cellulosilyticus*, *B*.*ovatus*, *B*. *uniformis*, *B*. *thetaiotaomicron*, *B*. *caccae*, *E*. *faecium*, *E*. *faecalis*, *E*. *infirmum*, *C*. *histolyticum*, *E*. *raffinosus*, *L*. *crispatus*, *A*. *radioresistens*, and *P*. *anaerobius*. Correlation was determined using the Pearson’s test.
(TIF)

S3 Fig LDH release from macrophages induced by commensal panel. LDH release from J774A.1 macrophages was measured using CyQUANT assay kit after macrophages were stimulated at an MOI of 1:10 for 24 hours. Data are expressed relative to positive control, n = 5 biological repeats.
(TIF)

S4 Fig Intestinal cytokine levels in antibiotic treated mice administered heat-killed consortia of Bacteroidetes or Firmicutes. IL6 cytokine levels 24 hours post oral inoculation with 1×10^8^ CFU of indicated heat-killed commensal consortium (BACTEROIDETES consortium: *Bacteroides caccae*, *Bacteroides cellulosilyticus*, *Bacteroides dorei*, *Bacteroides eggerthii*, *Bacteroides finegoldii*, *Bacteroides ovatus*, *Bacteroides thetaiotaomicron*, *Bacteroides uniformis*, *Parabacteroides johnsonii*; FIRMICUTES consortium: *Clostridium histolyticum*, *Clostridium orbiscindens*, *Enterococcus faecalis*, *Enterococcus raffinosus*, *Eubacterium limosum*, *Faecalibacterium prausnitzii*, *Lactobacillus reuteri*, *Lactobacillus crispatus*, *Paenibacillus barengoltzii*, *Peptostreptococcus anaerobius*). Each point represents a single mouse and horizontal lines indicate median values and statistical comparisons were by Mann Whitney test *p < 0.05.
(TIF)

S5 Fig Intestinal cytokine levels in germ-free mice after inoculation with either a consortium of Bacteroidetes or Firmicutes. Intestinal cytokine levels in germ-free mice 24 hours post oral inoculation with 1×10^8^ CFU of indicated commensal consortium (BACTEROIDETES consortium: *Bacteroides caccae*, *Bacteroides cellulosilyticus*, *Bacteroides dorei*, *Bacteroides eggerthii*, *Bacteroides finegoldii*, *Bacteroides ovatus*, *Bacteroides thetaiotaomicron*, *Bacteroides uniformis*, *Parabacteroides johnsonii*; FIRMICUTES consortium: *Clostridium histolyticum*, *Clostridium orbiscindens*, *Enterococcus faecalis*, *Enterococcus raffinosus*, *Eubacterium limosum*, *Faecalibacterium prausnitzii*, *Lactobacillus reuteri*, *Lactobacillus crispatus*, *Paenibacillus barengoltzii*, *Peptostreptococcus anaerobius*). Each point represents a single mouse and all mice were antibiotic treated unless indicated otherwise prior to commensal administration. Statistical comparisons were by Mann Whitney test, horizontal lines indicate median values, *p < 0.05, **p < 0.01, and NS, not significant.
(TIF)

S6 Fig Bacteroidetes and Firmicutes exert their effects on intestinal immunity independently of neutrophils. Intestinal cytokine levels hours post oral inoculation with 1×10^8^ CFU of indicated commensal consortium (BACTEROIDETES consortium: *Bacteroides caccae*, *Bacteroides cellulosilyticus*, *Bacteroides dorei*, *Bacteroides eggerthii*, *Bacteroides finegoldii*, *Bacteroides ovatus*, *Bacteroides thetaiotaomicron*, *Bacteroides uniformis*, *Parabacteroides johnsonii;* FIRMICUTES consortium: *Clostridium histolyticum*, *Clostridium orbiscindens*, *Enterococcus faecalis*, *Enterococcus raffinosus*, *Eubacterium limosum*, *Faecalibacterium prausnitzii*, *Lactobacillus reuteri*, *Lactobacillus crispatus*, *Paenibacillus barengoltzii*, *Peptostreptococcus anaerobius*). Indicated mice were treated with the neutrophil-depleting 1A8 antibody, or isotype control 3 and 1 days before consortia inoculation (100 μg/mouse via intraperitoneal injection). All mice were antibiotic treated unless indicated otherwise prior to commensal administration. Statistical comparisons were by Kruskal–Wallis test with Dunn’s multiple comparison test. *p < 0.05, **p < 0.01, and NS, not significant.
(TIF)

S7 Fig Gram-positive commensal stimulation of macrophages and TLR4 is not due to contaminating LPS. (a) J774A.1 macrophages were stimulated by indicated commensals at an MOI of 1:10, or LPS (100 ng/mL) for 24 hours with or without polymyxin B (25 μg/ml polymyxin B) and cytokine production then measured by ELISA. (b) TLR4-dependent SEAP production by HEK293 cells 24 hours post-stimulation with indicated commensal species at MOI of 1:10, or LPS (100 ng/mL). (c) J774A.1 macrophages were stimulated by indicated commensal culture media (10 μL), or LPS (100 ng/mL, the first bar) for 24 hours cytokine production then measured by ELISA. Data are expressed relative to no polymyxin B controls, n = 5 biological repeats. Statistical comparisons were by Mann Whitney test *p < 0.05, NS, not significant.
(TIF)

S8 Fig Reduction in cytokine production after IRAK4 inhibition. Mean reduction in macrophage IL6 production after inhibition of IRAK4. Statistical significance was determined by a two way ANOVA with post-hoc Turkey’s test comparing macrophages treated with 100 nM PF06650833 against macrophages treated with vehicle control (n = 4–10 biological repeats). *p < 0.05, **p < 0.01, ***p < 0.001 and NS, not significant.
(TIF)

S9 Fig siRNA-mediated confirmation that canonical NF-κB signalling is the ultimate calibrator of commensal immunostimulatory capacity. (a) Heat map displaying percentage inhibition in IL6 production induced by commensal stimulation after siRNA-mediated inhibition of JNK1 and JNK2, or IKKβ. Data are relative to macrophages treated with control siRNA, with mean values for percentage inhibition for each species are shown (n = 5 biological repeats, at MOI of 1:10). (b) Correlation between overall immunostimulatory capacity of all commensals, the Bacteroidetes, and Firmicutes in our panel (measured by macrophage IL6 production) and their dependence on canonical NF-κB signalling (measured by cognate reduction in cytokine production incurred after inhibition of canonical NF-κB signalling). Correlations were determined by Spearman’s test.
(TIF)

S10 Fig VRE burden in mice administered heat-killed commensal consortia. VRE burden in adult antibiotic-treated mice two days post-oral inoculation with 1×10^8^ CFU of indicated heat-killed commensal consortium two days prior to VRE. Each point represents a single mouse and horizontal lines indicate median values and statistical comparisons were by Mann Whitney test **p < 0.01.
(TIF)

S11 Fig Engraftment of the “low immune-stimulating” and “high immune-stimulating” consortia. (a) Total bacterial, Bacteroidetes and Firmicutes levels in faeces of mice WT adult mice orally inoculated with 1×10^8^ CFU of the “low immune-stimulating” commensal consortium, 1×10^8^ CFU of the “high immune-stimulating” commensal consortium two days prior to VRE inoculation. (b) Relative abundance of each commensal in faeces of mice WT adult mice orally inoculated with 1×10^8^ CFU of the “low immune-stimulating” commensal consortium. Levels of each species were determined by species-specific qPCR and related to total bacterial levels determined by qPCR. Fecal levels of viable Lactobacilli were determined by culture on MRS media, Bacteroidetes by culture on BBE media, and Clostridia by culture on MRCA (with Bacteroidetes level subtracted as MRCA can support growth of Bacteroidetes as well as Clostridia). (c) Relative abundance of each commensal in faeces of WT adult mice orally inoculated with 1×10^8^ CFU of the “high immune-stimulating” commensal consortium two days prior to VRE inoculation. Levels of each species were determined by species-specific qPCR and related to total bacterial levels determined by qPCR. Fecal levels of viable Bacteroidetes were determined by culture on BBE media, and Clostridia by culture on MRCA (with Bacteroidetes level subtracted as MRCA can support growth of Bacteroidetes as well as Clostridia), and *P*. *anaerobius* by culture on TSA media +/- Sodium Polyanethol Sulfonate at 0.05% (as sensitivity to Sodium Polyanethol Sulfonate is used to identify *P*. *anaerobius* and other consortium members are insensitive to it). For relative abundance each bar represents the means of n = 5 animals.
(TIF)

S12 Fig Clodronate depletes intestinal macrophages intestine. The expression of the macrophage marker *F4/80* was measured in the intestinal tissue of antibiotic treated mice after clodronate liposomes or empty liposome treatment (n = 5 animals). Statistical comparisons were by Mann Whitney, horizontal lines indicate median values, **p < 0.01.
(TIF)

S13 Fig 1A8 treatment depletes intestinal neutrophils. MPO levels as a marker for neutrophil levels were measured in the intestinal tissue of antibiotic treated mice after 1A8 treatment or isotype control (100 μg/mouse via intraperitoneal injection). Mice were treated with the neutrophil-depleting 1A8 antibody, or isotype control 3 and 1 days before consortia inoculation. (n = 5 animals). Statistical comparisons were by Mann Whitney, horizontal lines indicate median values, **p < 0.01.
(TIF)

S1 Table Bacterial species used in this study and their growth conditions. BHIS = supplemented brain heart infusion; CMM = chopped meat media; MRS = DeMan, Rogosa and Sharpe broth; CDB = Clostrida differential broth; TSB = tryptic soya broth; MRCB = modified reinforced clostridial broth; LB = Luria-Bertani broth; BHI = brain heart infusion.
(TIF)

## References

[1] Brown RL, Clarke TB. The regulation of host defences to infection by the microbiota. Immunology. 2017;150(1):1–6. 10.1111/imm.12634 27311879 PMC5221693

[2] Honda K, Littman DR. The microbiome in infectious disease and inflammation. Annu Rev Immunol. 2012;30:759–95. 10.1146/annurev-immunol-020711-074937 22224764 PMC4426968

[3] Kawai T, Akira S. The role of pattern-recognition receptors in innate immunity: update on Toll-like receptors. Nat Immunol. 2010;11(5):373–84. 10.1038/ni.1863 20404851

[4] Baumler AJ, Sperandio V. Interactions between the microbiota and pathogenic bacteria in the gut. Nature. 2016;535(7610):85–93. 10.1038/nature18849 27383983 PMC5114849

[5] Cho I, Blaser MJ. The human microbiome: at the interface of health and disease. Nat Rev Genet. 2012;13(4):260–70. 10.1038/nrg3182 22411464 PMC3418802

[6] Human Microbiome Project C. Structure, function and diversity of the healthy human microbiome. Nature. 2012;486(7402):207–14. 10.1038/nature11234 22699609 PMC3564958

[7] Keith JW, Pamer EG. Enlisting commensal microbes to resist antibiotic-resistant pathogens. J Exp Med. 2019;216(1):10–9. 10.1084/jem.20180399 30309968 PMC6314519

[8] Skelly AN, Sato Y, Kearney S, Honda K. Mining the microbiota for microbial and metabolite-based immunotherapies. Nat Rev Immunol. 2019;19(5):305–23. 10.1038/s41577-019-0144-5 30858494

[9] Chu H, Mazmanian SK. Innate immune recognition of the microbiota promotes host-microbial symbiosis. Nat Immunol. 2013;14(7):668–75. 10.1038/ni.2635 23778794 PMC4109969

[10] Clarke TB. Microbial programming of systemic innate immunity and resistance to infection. PLoS Pathog. 2014;10(12):e1004506 10.1371/journal.ppat.1004506 25474680 PMC4256436

[11] Rakoff-Nahoum S, Paglino J, Eslami-Varzaneh F, Edberg S, Medzhitov R. Recognition of commensal microflora by toll-like receptors is required for intestinal homeostasis. Cell. 2004;118(2):229–41. 10.1016/j.cell.2004.07.002 15260992

[12] Oh JZ, Ravindran R, Chassaing B, Carvalho FA, Maddur MS, Bower M, et al TLR5-mediated sensing of gut microbiota is necessary for antibody responses to seasonal influenza vaccination. Immunity. 2014;41(3):478–92. 10.1016/j.immuni.2014.08.009 25220212 PMC4169736

[13] Brown RL, Sequeira RP, Clarke TB. The microbiota protects against respiratory infection via GM-CSF signaling. Nat Commun. 2017;8(1):1512 10.1038/s41467-017-01803-x 29142211 PMC5688119

[14] Deshmukh HS, Liu Y, Menkiti OR, Mei J, Dai N, O'Leary CE, et al The microbiota regulates neutrophil homeostasis and host resistance to Escherichia coli K1 sepsis in neonatal mice. Nat Med. 2014;20(5):524–30. 10.1038/nm.3542 24747744 PMC4016187

[15] Clarke TB, Davis KM, Lysenko ES, Zhou AY, Yu Y, Weiser JN. Recognition of peptidoglycan from the microbiota by Nod1 enhances systemic innate immunity. Nat Med. 2010;16(2):228–31. 10.1038/nm.2087 20081863 PMC4497535

[16] Round JL, Lee SM, Li J, Tran G, Jabri B, Chatila TA, et al The Toll-like receptor 2 pathway establishes colonization by a commensal of the human microbiota. Science. 2011;332(6032):974–7. 10.1126/science.1206095 21512004 PMC3164325

[17] Balmer ML, Schurch CM, Saito Y, Geuking MB, Li H, Cuenca M, et al Microbiota-derived compounds drive steady-state granulopoiesis via MyD88/TICAM signaling. J Immunol. 2014;193(10):5273–83. 10.4049/jimmunol.1400762 25305320

[18] Karmarkar D, Rock KL. Microbiota signalling through MyD88 is necessary for a systemic neutrophilic inflammatory response. Immunology. 2013;140(4):483–92. 10.1111/imm.12159 23909393 PMC3839652

[19] Hergott CB, Roche AM, Tamashiro E, Clarke TB, Bailey AG, Laughlin A, et al Peptidoglycan from the gut microbiota governs the lifespan of circulating phagocytes at homeostasis. Blood. 2016;127(20):2460–71. 10.1182/blood-2015-10-675173 26989200 PMC4874226

[20] Brandl K, Plitas G, Mihu CN, Ubeda C, Jia T, Fleisher M, et al Vancomycin-resistant enterococci exploit antibiotic-induced innate immune deficits. Nature. 2008;455(7214):804–7. 10.1038/nature07250 18724361 PMC2663337

[21] Gomez de Aguero M, Ganal-Vonarburg SC, Fuhrer T, Rupp S, Uchimura Y, Li H, et al The maternal microbiota drives early postnatal innate immune development. Science. 2016;351(6279):1296–302. 10.1126/science.aad2571 26989247

[22] Akira S, Takeda K. Toll-like receptor signalling. Nat Rev Immunol. 2004;4(7):499–511. 10.1038/nri1391 15229469

[23] O'Toole PW, Marchesi JR, Hill C. Next-generation probiotics: the spectrum from probiotics to live biotherapeutics. Nat Microbiol. 2017;2:17057 10.1038/nmicrobiol.2017.57 28440276

[24] Banerjee S, Schlaeppi K, van der Heijden MGA. Keystone taxa as drivers of microbiome structure and functioning. Nat Rev Microbiol. 2018;16(9):567–76. 10.1038/s41579-018-0024-1 29789680

[25] Thaiss CA, Zmora N, Levy M, Elinav E. The microbiome and innate immunity. Nature. 2016;535(7610):65–74. 10.1038/nature18847 27383981

[26] Nakagaki BN, Vieira AT, Rezende RM, David BA, Menezes GB. Tissue macrophages as mediators of a healthy relationship with gut commensal microbiota. Cell Immunol. 2018;330:16–26. 10.1016/j.cellimm.2018.01.017 29422270

[27] Bain CC, Mowat AM. Macrophages in intestinal homeostasis and inflammation. Immunol Rev. 2014;260(1):102–17. 10.1111/imr.12192 24942685 PMC4141699

[28] Gordon S, Pluddemann A. Tissue macrophages: heterogeneity and functions. BMC Biol. 2017;15(1):53 10.1186/s12915-017-0392-4 28662662 PMC5492929

[29] Foster SL, Hargreaves DC, Medzhitov R. Gene-specific control of inflammation by TLR-induced chromatin modifications. Nature. 2007;447(7147):972–8. 10.1038/nature05836 17538624

[30] Ramirez-Carrozzi VR, Braas D, Bhatt DM, Cheng CS, Hong C, Doty KR, et al A unifying model for the selective regulation of inducible transcription by CpG islands and nucleosome remodeling. Cell. 2009;138(1):114–28. 10.1016/j.cell.2009.04.020 19596239 PMC2712736

[31] Tebbutt NC, Giraud AS, Inglese M, Jenkins B, Waring P, Clay FJ, et al Reciprocal regulation of gastrointestinal homeostasis by SHP2 and STAT-mediated trefoil gene activation in gp130 mutant mice. Nat Med. 2002;8(10):1089–97. 10.1038/nm763 12219085

[32] Bradford EM, Ryu SH, Singh AP, Lee G, Goretsky T, Sinh P, et al Epithelial TNF Receptor Signaling Promotes Mucosal Repair in Inflammatory Bowel Disease. J Immunol. 2017;199(5):1886–97. 10.4049/jimmunol.1601066 28747340 PMC5568528

[33] Tong AJ, Liu X, Thomas BJ, Lissner MM, Baker MR, Senagolage MD, et al A Stringent Systems Approach Uncovers Gene-Specific Mechanisms Regulating Inflammation. Cell. 2016;165(1):165–79. 10.1016/j.cell.2016.01.020 26924576 PMC4808443

[34] Kawai T, Akira S. Signaling to NF-kappaB by Toll-like receptors. Trends Mol Med. 2007;13(11):460–9. 10.1016/j.molmed.2007.09.002 18029230

[35] Lin B, Dutta B, Fraser IDC. Systematic Investigation of Multi-TLR Sensing Identifies Regulators of Sustained Gene Activation in Macrophages. Cell Syst. 2017;5(1):25–37 e3. 10.1016/j.cels.2017.06.014 28750197 PMC5584636

[36] Gottschalk RA, Martins AJ, Angermann BR, Dutta B, Ng CE, Uderhardt S, et al Distinct NF-kappaB and MAPK Activation Thresholds Uncouple Steady-State Microbe Sensing from Anti-pathogen Inflammatory Responses. Cell Syst. 2016;2(6):378–90. 10.1016/j.cels.2016.04.016 27237739 PMC4919147

[37] Kim TW, Staschke K, Bulek K, Yao J, Peters K, Oh KH, et al A critical role for IRAK4 kinase activity in Toll-like receptor-mediated innate immunity. J Exp Med. 2007;204(5):1025–36. 10.1084/jem.20061825 17470642 PMC2118590

[38] Clark K. Protein kinase networks that limit TLR signalling. Biochem Soc Trans. 2014;42(1):11–24. 10.1042/BST20130124 24450622

[39] Loegering DJ, Lennartz MR. Protein kinase C and toll-like receptor signaling. Enzyme Res. 2011;2011:537821 10.4061/2011/537821 21876792 PMC3162977

[40] Chattopadhyay S, Sen GC. Tyrosine phosphorylation in Toll-like receptor signaling. Cytokine Growth Factor Rev. 2014;25(5):533–41. 10.1016/j.cytogfr.2014.06.002 25022196 PMC4254339

[41] Arthur JS, Ley SC. Mitogen-activated protein kinases in innate immunity. Nat Rev Immunol. 2013;13(9):679–92. 10.1038/nri3495 23954936

[42] Pamer EG. Resurrecting the intestinal microbiota to combat antibiotic-resistant pathogens. Science. 2016;352(6285):535–8. 10.1126/science.aad9382 27126035 PMC4984266

[43] Boucher HW, Talbot GH, Bradley JS, Edwards JE, Gilbert D, Rice LB, et al Bad bugs, no drugs: no ESKAPE! An update from the Infectious Diseases Society of America. Clin Infect Dis. 2009;48(1):1–12. 10.1086/595011 19035777

[44] Sequeira RP, McDonald JAK, Marchesi JR, Clarke TB. Commensal Bacteroidetes protect against Klebsiella pneumoniae colonization and transmission through IL-36 signalling. Nat Microbiol. 2020 10.1038/s41564-019-0640-1 31907407 PMC7610889

[45] Janssens Y, Nielandt J, Bronselaer A, Debunne N, Verbeke F, Wynendaele E, et al Disbiome database: linking the microbiome to disease. BMC Microbiol. 2018;18(1):50 10.1186/s12866-018-1197-5 29866037 PMC5987391

[46] Rath HC, Herfarth HH, Ikeda JS, Grenther WB, Hamm TE, Jr., Balish E, et al Normal luminal bacteria, especially Bacteroides species, mediate chronic colitis, gastritis, and arthritis in HLA-B27/human beta2 microglobulin transgenic rats. J Clin Invest. 1996;98(4):945–53. 10.1172/JCI118878 8770866 PMC507509

[47] Lucke K, Miehlke S, Jacobs E, Schuppler M. Prevalence of Bacteroides and Prevotella spp. in ulcerative colitis. J Med Microbiol. 2006;55(Pt 5):617–24. 10.1099/jmm.0.46198-0 16585651

[48] Mariat D, Firmesse O, Levenez F, Guimaraes V, Sokol H, Dore J, et al The Firmicutes/Bacteroidetes ratio of the human microbiota changes with age. BMC Microbiol. 2009;9:123 10.1186/1471-2180-9-123 19508720 PMC2702274

[49] Ley RE, Turnbaugh PJ, Klein S, Gordon JI. Microbial ecology: human gut microbes associated with obesity. Nature. 2006;444(7122):1022–3. 10.1038/4441022a 17183309

[50] Jacobson AN, Choudhury BP, Fischbach MA. The Biosynthesis of Lipooligosaccharide from Bacteroides thetaiotaomicron. MBio. 2018;9(2). 10.1128/mBio.02289-17 29535205 PMC5850320

[51] Maskell JP. The resolution of bacteroides lipopolysaccharides by polyacrylamide gel electrophoresis. J Med Microbiol. 1991;34(5):253–7. 10.1099/00222615-34-5-253 2030500

[52] Maskell JP. Electrophoretic analysis of the lipopolysaccharides of Bacteroides spp. Antonie Van Leeuwenhoek. 1994;65(2):155–61. 10.1007/BF00871756 7979320

[53] Ryan A, Lynch M, Smith SM, Amu S, Nel HJ, McCoy CE, et al A role for TLR4 in Clostridium difficile infection and the recognition of surface layer proteins. PLoS Pathog. 2011;7(6):e1002076 10.1371/journal.ppat.1002076 21738466 PMC3128122

[54] Alameddine J, Godefroy E, Papargyris L, Sarrabayrouse G, Tabiasco J, Bridonneau C, et al Faecalibacterium prausnitzii Skews Human DC to Prime IL10-Producing T Cells Through TLR2/6/JNK Signaling and IL-10, IL-27, CD39, and IDO-1 Induction. Front Immunol. 2019;10:143 10.3389/fimmu.2019.00143 30787928 PMC6373781

[55] Sui SJ, Tian ZB, Wang QC, Chen R, Nie J, Li JS, et al Clostridium butyricum promotes intestinal motility by regulation of TLR2 in interstitial cells of Cajal. Eur Rev Med Pharmacol Sci. 2018;22(14):4730–8. 10.26355/eurrev_201807_15533 30058712

[56] Bianchi ME. DAMPs, PAMPs and alarmins: all we need to know about danger. J Leukoc Biol. 2007;81(1):1–5. 10.1189/jlb.0306164 17032697

[57] Beutler B. Neo-ligands for innate immune receptors and the etiology of sterile inflammatory disease. Immunol Rev. 2007;220:113–28. 10.1111/j.1600-065X.2007.00577.x 17979843

[58] Takeuchi O, Hoshino K, Kawai T, Sanjo H, Takada H, Ogawa T, et al Differential roles of TLR2 and TLR4 in recognition of gram-negative and gram-positive bacterial cell wall components. Immunity. 1999;11(4):443–51. 10.1016/s1074-7613(00)80119-3 10549626

[59] Chu M, Zhou M, Jiang C, Chen X, Guo L, Zhang M, et al Staphylococcus aureus Phenol-Soluble Modulins alpha1-alpha3 Act as Novel Toll-Like Receptor (TLR) 4 Antagonists to Inhibit HMGB1/TLR4/NF-kappaB Signaling Pathway. Front Immunol. 2018;9:862 10.3389/fimmu.2018.00862 29922279 PMC5996891

[60] Park JM, Ng VH, Maeda S, Rest RF, Karin M. Anthrolysin O and other gram-positive cytolysins are toll-like receptor 4 agonists. J Exp Med. 2004;200(12):1647–55. 10.1084/jem.20041215 15611291 PMC2211988

[61] Netea MG, Gow NA, Munro CA, Bates S, Collins C, Ferwerda G, et al Immune sensing of Candida albicans requires cooperative recognition of mannans and glucans by lectin and Toll-like receptors. J Clin Invest. 2006;116(6):1642–50. 10.1172/JCI27114 16710478 PMC1462942

[62] Shoham S, Huang C, Chen JM, Golenbock DT, Levitz SM. Toll-like receptor 4 mediates intracellular signaling without TNF-alpha release in response to Cryptococcus neoformans polysaccharide capsule. J Immunol. 2001;166(7):4620–6. 10.4049/jimmunol.166.7.4620 11254720

[63] Wang Y, Su L, Morin MD, Jones BT, Whitby LR, Surakattula MM, et al TLR4/MD-2 activation by a synthetic agonist with no similarity to LPS. Proc Natl Acad Sci U S A. 2016;113(7):E884–93. 10.1073/pnas.1525639113 26831104 PMC4763747

[64] Sun J, Li N, Oh KS, Dutta B, Vayttaden SJ, Lin B, et al Comprehensive RNAi-based screening of human and mouse TLR pathways identifies species-specific preferences in signaling protein use. Sci Signal. 2016;9(409):ra3 10.1126/scisignal.aab2191 26732763 PMC5381726

[65] Bain CC, Bravo-Blas A, Scott CL, Perdiguero EG, Geissmann F, Henri S, et al Constant replenishment from circulating monocytes maintains the macrophage pool in the intestine of adult mice. Nat Immunol. 2014;15(10):929–37. 10.1038/ni.2967 25151491 PMC4169290

[66] Bain CC, Scott CL, Uronen-Hansson H, Gudjonsson S, Jansson O, Grip O, et al Resident and pro-inflammatory macrophages in the colon represent alternative context-dependent fates of the same Ly6Chi monocyte precursors. Mucosal Immunol. 2013;6(3):498–510. 10.1038/mi.2012.89 22990622 PMC3629381

[67] Bain CC, Oliphant CJ, Thomson CA, Kullberg MC, Mowat AM. Proinflammatory Role of Monocyte-Derived CX3CR1(int) Macrophages in Helicobacter hepaticus-Induced Colitis. Infect Immun. 2018;86(2). 10.1128/IAI.00579-17 PMC5778360 29203542

[68] Weber B, Saurer L, Schenk M, Dickgreber N, Mueller C. CX3CR1 defines functionally distinct intestinal mononuclear phagocyte subsets which maintain their respective functions during homeostatic and inflammatory conditions. Eur J Immunol. 2011;41(3):773–9. 10.1002/eji.201040965 21341263

[69] Chang PV, Hao L, Offermanns S, Medzhitov R. The microbial metabolite butyrate regulates intestinal macrophage function via histone deacetylase inhibition. Proc Natl Acad Sci U S A. 2014;111(6):2247–52. 10.1073/pnas.1322269111 24390544 PMC3926023

[70] Sansonetti PJ, Medzhitov R. Learning tolerance while fighting ignorance. Cell. 2009;138(3):416–20. 10.1016/j.cell.2009.07.024 19665961

[71] Hoshi N, Schenten D, Nish SA, Walther Z, Gagliani N, Flavell RA, et al MyD88 signalling in colonic mononuclear phagocytes drives colitis in IL-10-deficient mice. Nat Commun. 2012;3:1120 10.1038/ncomms2113 23047678 PMC3521499

[72] Kuhn KA, Schulz HM, Regner EH, Severs EL, Hendrickson JD, Mehta G, et al Bacteroidales recruit IL-6-producing intraepithelial lymphocytes in the colon to promote barrier integrity. Mucosal Immunol. 2018;11(2):357–68. 10.1038/mi.2017.55 28812548 PMC5815964

[73] Hudson LE, Anderson SE, Corbett AH, Lamb TJ. Gleaning Insights from Fecal Microbiota Transplantation and Probiotic Studies for the Rational Design of Combination Microbial Therapies. Clin Microbiol Rev. 2017;30(1):191–231. 10.1128/CMR.00049-16 27856521 PMC5217792

[74] Sorbara MT, Dubin K, Littmann ER, Moody TU, Fontana E, Seok R, et al Inhibiting antibiotic-resistant Enterobacteriaceae by microbiota-mediated intracellular acidification. J Exp Med. 2019;216(1):84–98. 10.1084/jem.20181639 30563917 PMC6314524

[75] Thiemann S, Smit N, Roy U, Lesker TR, Galvez EJC, Helmecke J, et al Enhancement of IFNgamma Production by Distinct Commensals Ameliorates Salmonella-Induced Disease. Cell Host Microbe. 2017;21(6):682–94 e5. 10.1016/j.chom.2017.05.005 28618267

[76] Jacobson A, Lam L, Rajendram M, Tamburini F, Honeycutt J, Pham T, et al A Gut Commensal-Produced Metabolite Mediates Colonization Resistance to Salmonella Infection. Cell Host Microbe. 2018;24(2):296–307 e7. 10.1016/j.chom.2018.07.002 30057174 PMC6223613

[77] Caballero S, Kim S, Carter RA, Leiner IM, Susac B, Miller L, et al Cooperating Commensals Restore Colonization Resistance to Vancomycin-Resistant Enterococcus faecium. Cell Host Microbe. 2017;21(5):592–602 e4. 10.1016/j.chom.2017.04.002 28494240 PMC5494988

[78] Lane ER, Zisman TL, Suskind DL. The microbiota in inflammatory bowel disease: current and therapeutic insights. J Inflamm Res. 2017;10:63–73. 10.2147/JIR.S116088 28652796 PMC5473501

[79] Hayden MS, Ghosh S. NF-kappaB, the first quarter-century: remarkable progress and outstanding questions. Genes Dev. 2012;26(3):203–34. 10.1101/gad.183434.111 22302935 PMC3278889

[80] Clarke TB. Early innate immunity to bacterial infection in the lung is regulated systemically by the commensal microbiota via nod-like receptor ligands. Infect Immun. 2014;82(11):4596–606. 10.1128/IAI.02212-14 25135683 PMC4249320

[81] Graves MH, Morello JA, Kocka FE. Sodium polyanethol sulfonate sensitivity of anaerobic cocci. Appl Microbiol. 1974;27(6):1131–3. 4598436 10.1128/am.27.6.1131-1133.1974 PMC380222

[82] Benjamini Y, Hochberg Y. Controlling the False Discovery Rate—a Practical and Powerful Approach to Multiple Testing. J R Stat Soc B. 1995;57(1):289–300.

