# Supplementary figures and images for: Immunological design of commensal communities to treat intestinal infection and inflammation
Source: PLoS Pathog. 2021 Jan 19;17(1):e1009191. doi: 10.1371/journal.ppat.1009191 (PMC7846104; doi:10.1371/journal.ppat.1009191)

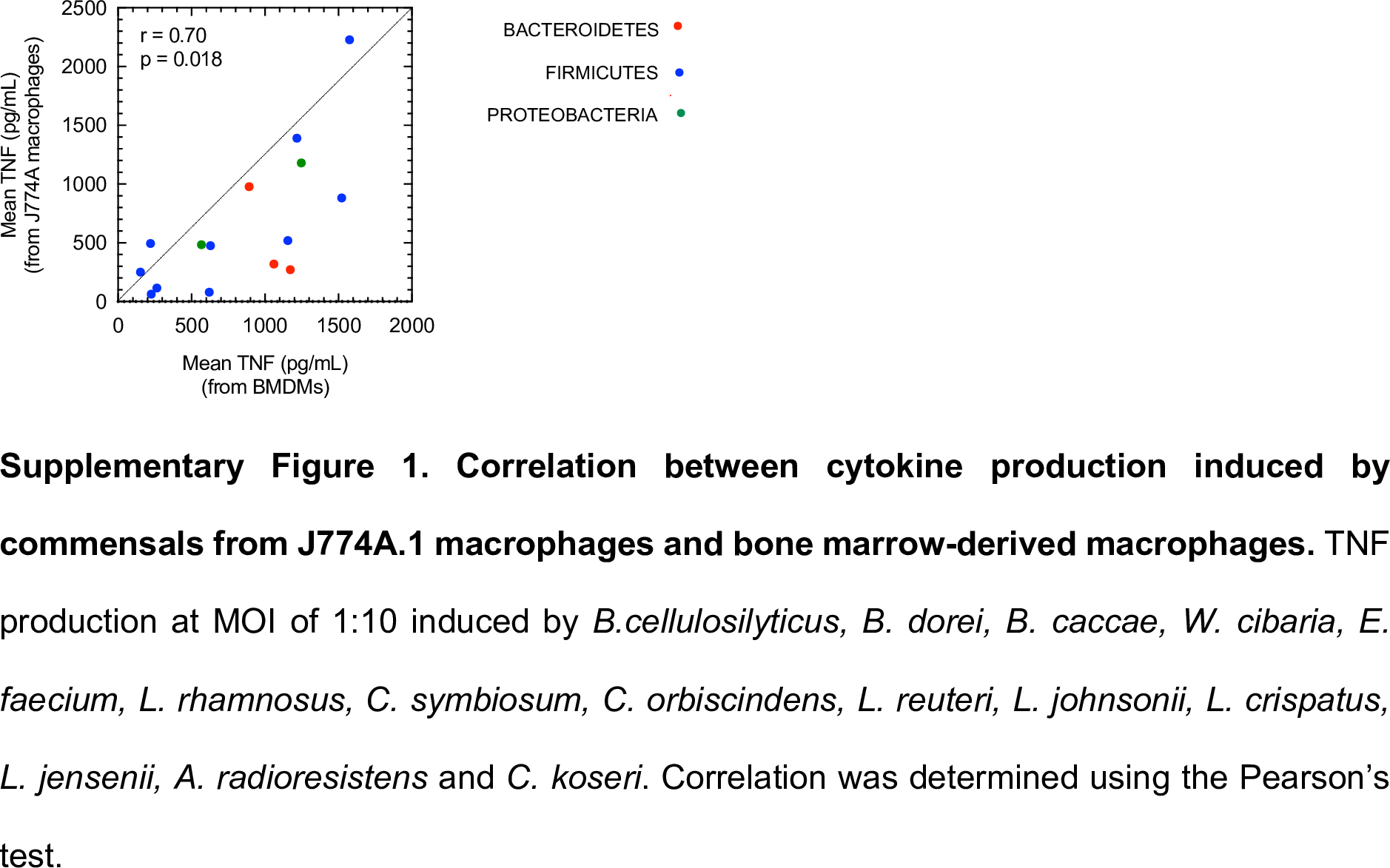

Supplement: S1 Fig — TNF production at MOI of 1:10 induced by B.cellulosilyticus, B. dorei, B. caccae, W. cibaria, E. faecium, L. rhamnosus, C. symbiosum, C. orbiscindens, L. reuteri, L. johnsonii, L. crispatus, L. jensenii, A. radioresistens and C. koseri. Correlation was determined using the Pearson’s test. (TIF) [file ppat.1009191.s001.tif]

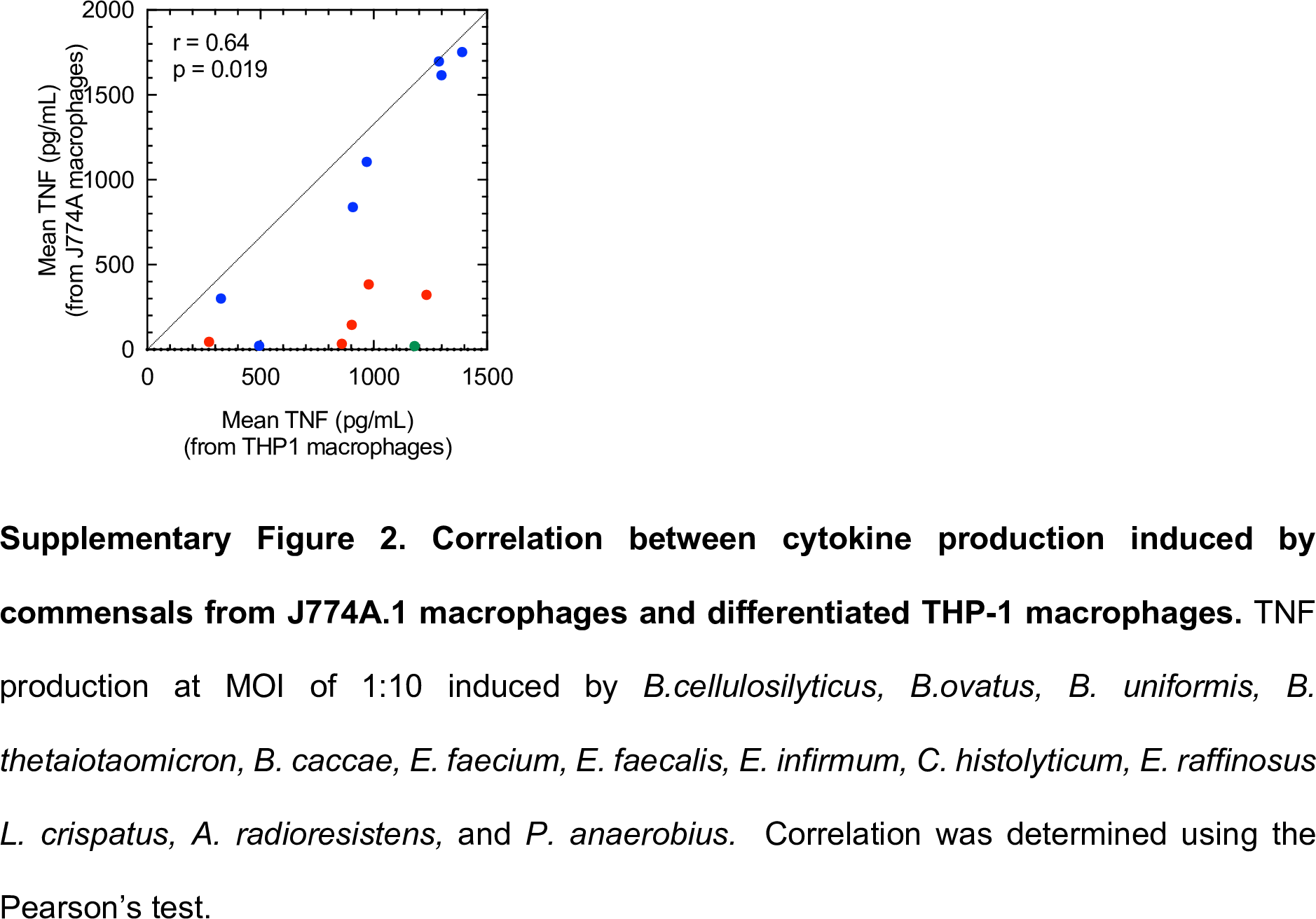

Supplement: S2 Fig — TNF production at MOI of 1:10 induced by B.cellulosilyticus, B.ovatus, B. uniformis, B. thetaiotaomicron, B. caccae, E. faecium, E. faecalis, E. infirmum, C. histolyticum, E. raffinosus, L. crispatus, A. radioresistens, and P. anaerobius. Correlation was determined using the Pearson’s test. (TIF) [file ppat.1009191.s002.tif]

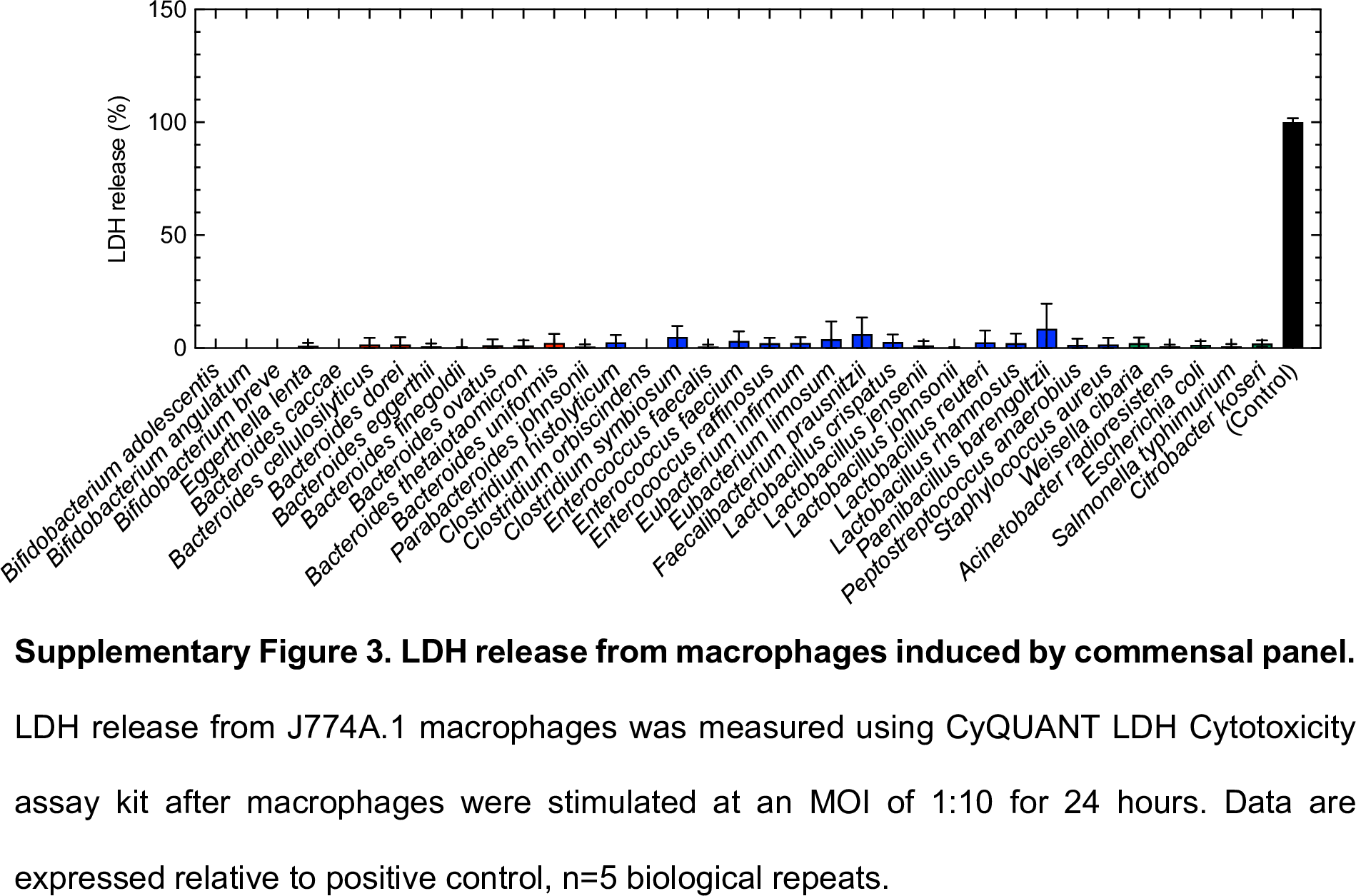

Supplement: S3 Fig — LDH release from J774A.1 macrophages was measured using CyQUANT assay kit after macrophages were stimulated at an MOI of 1:10 for 24 hours. Data are expressed relative to positive control, n = 5 biological repeats. (TIF) [file ppat.1009191.s003.tif]

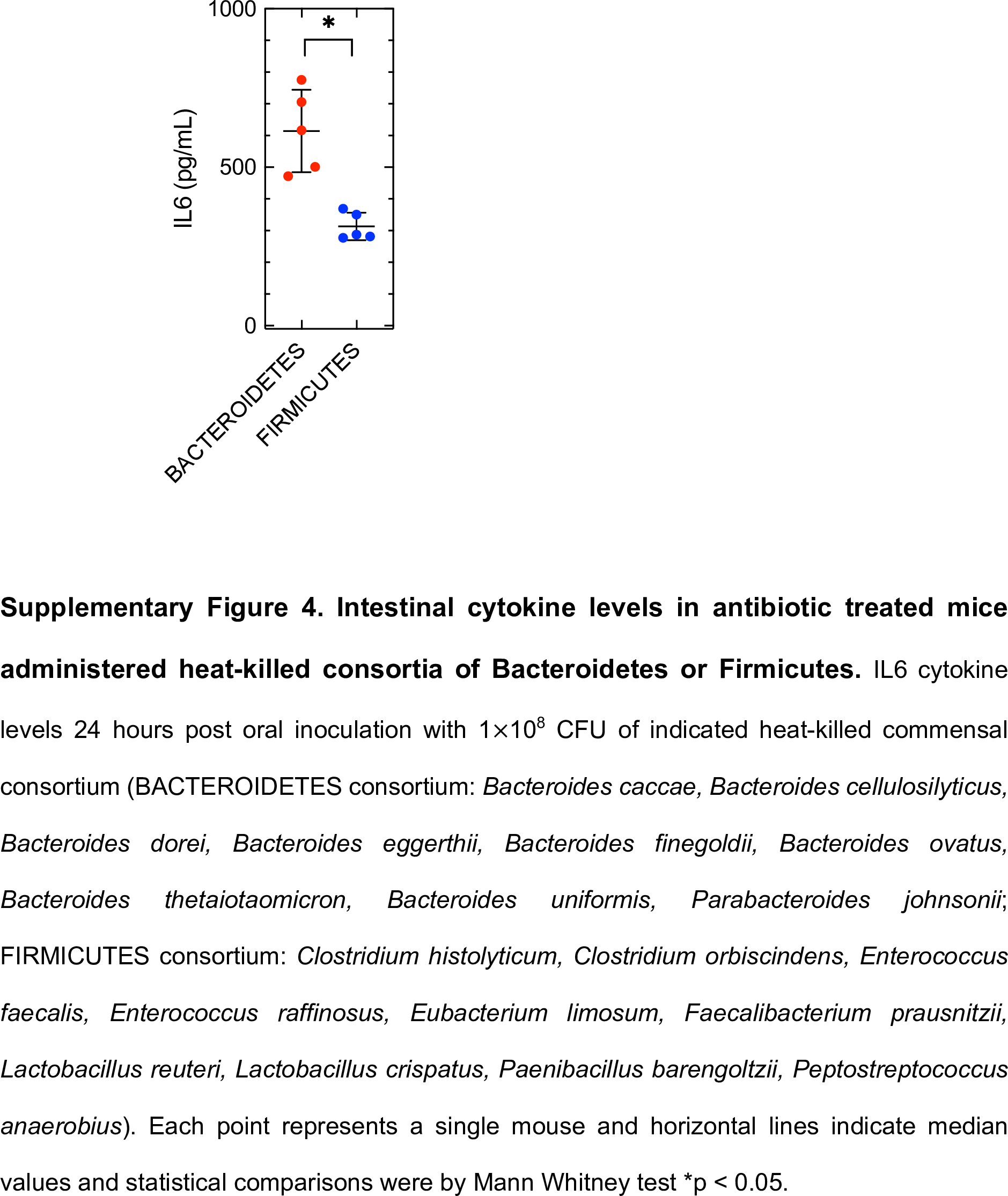

Supplement: S4 Fig — IL6 cytokine levels 24 hours post oral inoculation with 1×10^8^ CFU of indicated heat-killed commensal consortium (BACTEROIDETES consortium: Bacteroides caccae, Bacteroides cellulosilyticus, Bacteroides dorei, Bacteroides eggerthii, Bacteroides finegoldii, Bacteroides ovatus, Bacteroides thetaiotaomicron, Bacteroides uniformis, Parabacteroides johnsonii; FIRMICUTES consortium: Clostridium histolyticum, Clostridium orbiscindens, Enterococcus faecalis, Enterococcus raffinosus, Eubacterium limosum, Faecalibacterium prausnitzii, Lactobacillus reuteri, Lactobacillus crispatus, Paenibacillus barengoltzii, Peptostreptococcus anaerobius). Each point represents a single mouse and horizontal lines indicate median values and statistical comparisons were by Mann Whitney test *p < 0.05. (TIF) [file ppat.1009191.s004.tif]

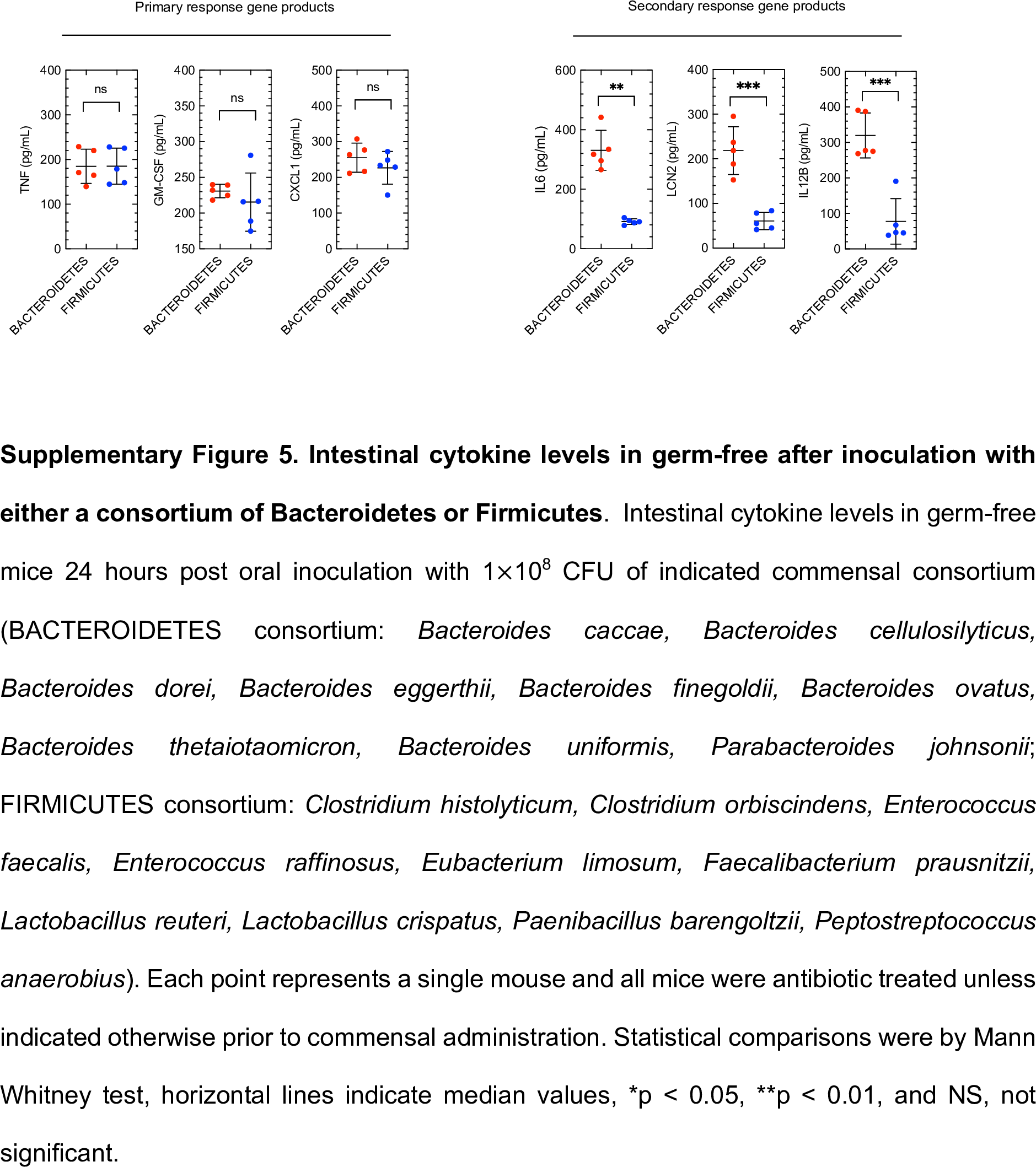

Supplement: S5 Fig — Intestinal cytokine levels in germ-free mice 24 hours post oral inoculation with 1×10^8^ CFU of indicated commensal consortium (BACTEROIDETES consortium: Bacteroides caccae, Bacteroides cellulosilyticus, Bacteroides dorei, Bacteroides eggerthii, Bacteroides finegoldii, Bacteroides ovatus, Bacteroides thetaiotaomicron, Bacteroides uniformis, Parabacteroides johnsonii; FIRMICUTES consortium: Clostridium histolyticum, Clostridium orbiscindens, Enterococcus faecalis, Enterococcus raffinosus, Eubacterium limosum, Faecalibacterium prausnitzii, Lactobacillus reuteri, Lactobacillus crispatus, Paenibacillus barengoltzii, Peptostreptococcus anaerobius). Each point represents a single mouse and all mice were antibiotic treated unless indicated otherwise prior to commensal administration. Statistical comparisons were by Mann Whitney test, horizontal lines indicate median values, *p < 0.05, **p < 0.01, and NS, not significant. (TIF) [file ppat.1009191.s005.tif]

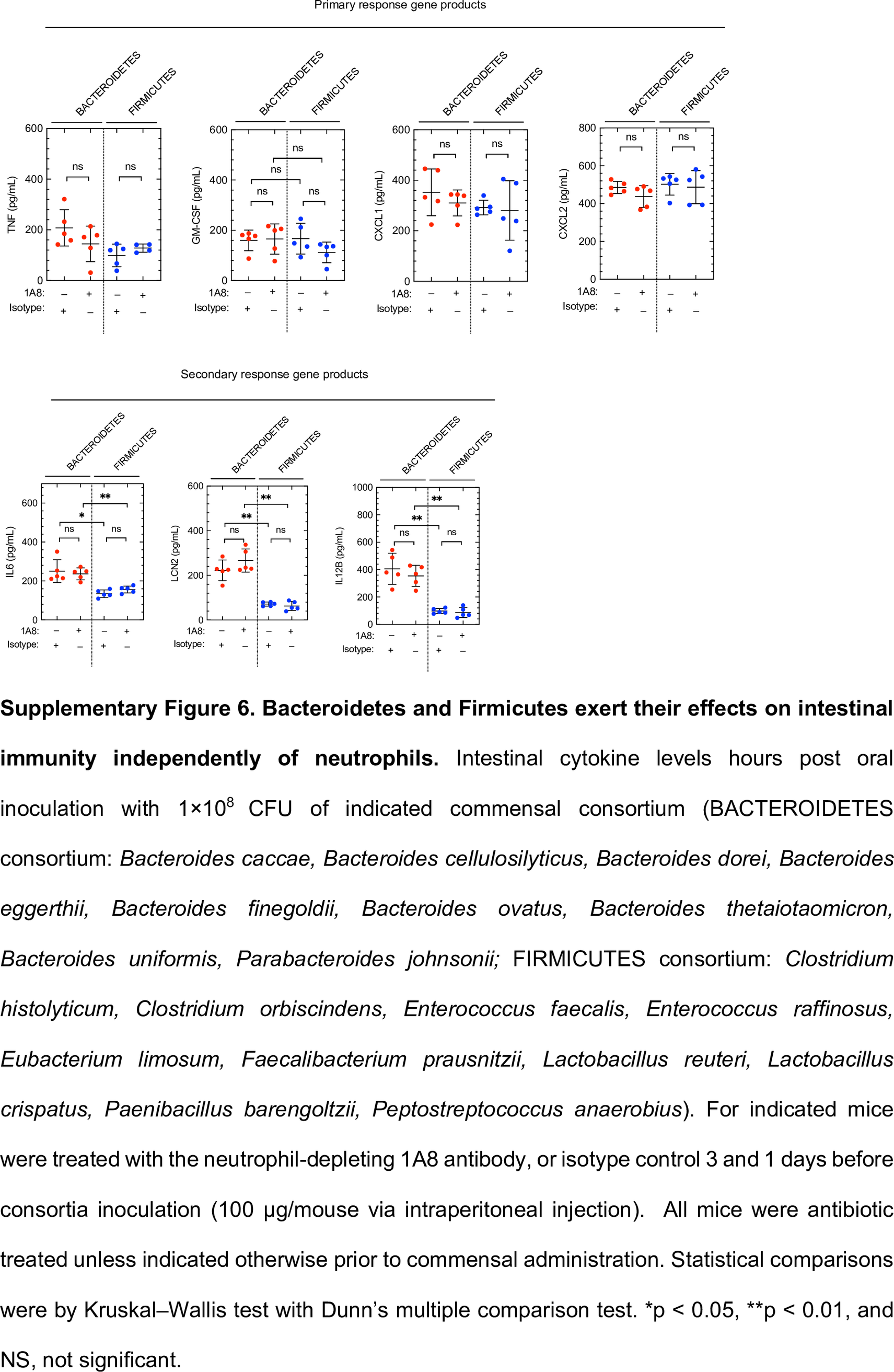

Supplement: S6 Fig — Intestinal cytokine levels hours post oral inoculation with 1×10^8^ CFU of indicated commensal consortium (BACTEROIDETES consortium: Bacteroides caccae, Bacteroides cellulosilyticus, Bacteroides dorei, Bacteroides eggerthii, Bacteroides finegoldii, Bacteroides ovatus, Bacteroides thetaiotaomicron, Bacteroides uniformis, Parabacteroides johnsonii; FIRMICUTES consortium: Clostridium histolyticum, Clostridium orbiscindens, Enterococcus faecalis, Enterococcus raffinosus, Eubacterium limosum, Faecalibacterium prausnitzii, Lactobacillus reuteri, Lactobacillus crispatus, Paenibacillus barengoltzii, Peptostreptococcus anaerobius). Indicated mice were treated with the neutrophil-depleting 1A8 antibody, or isotype control 3 and 1 days before consortia inoculation (100 μg/mouse via intraperitoneal injection). All mice were antibiotic treated unless indicated otherwise prior to commensal administration. Statistical comparisons were by Kruskal–Wallis test with Dunn’s multiple comparison test. *p < 0.05, **p < 0.01, and NS, not significant. (TIF) [file ppat.1009191.s006.tif]

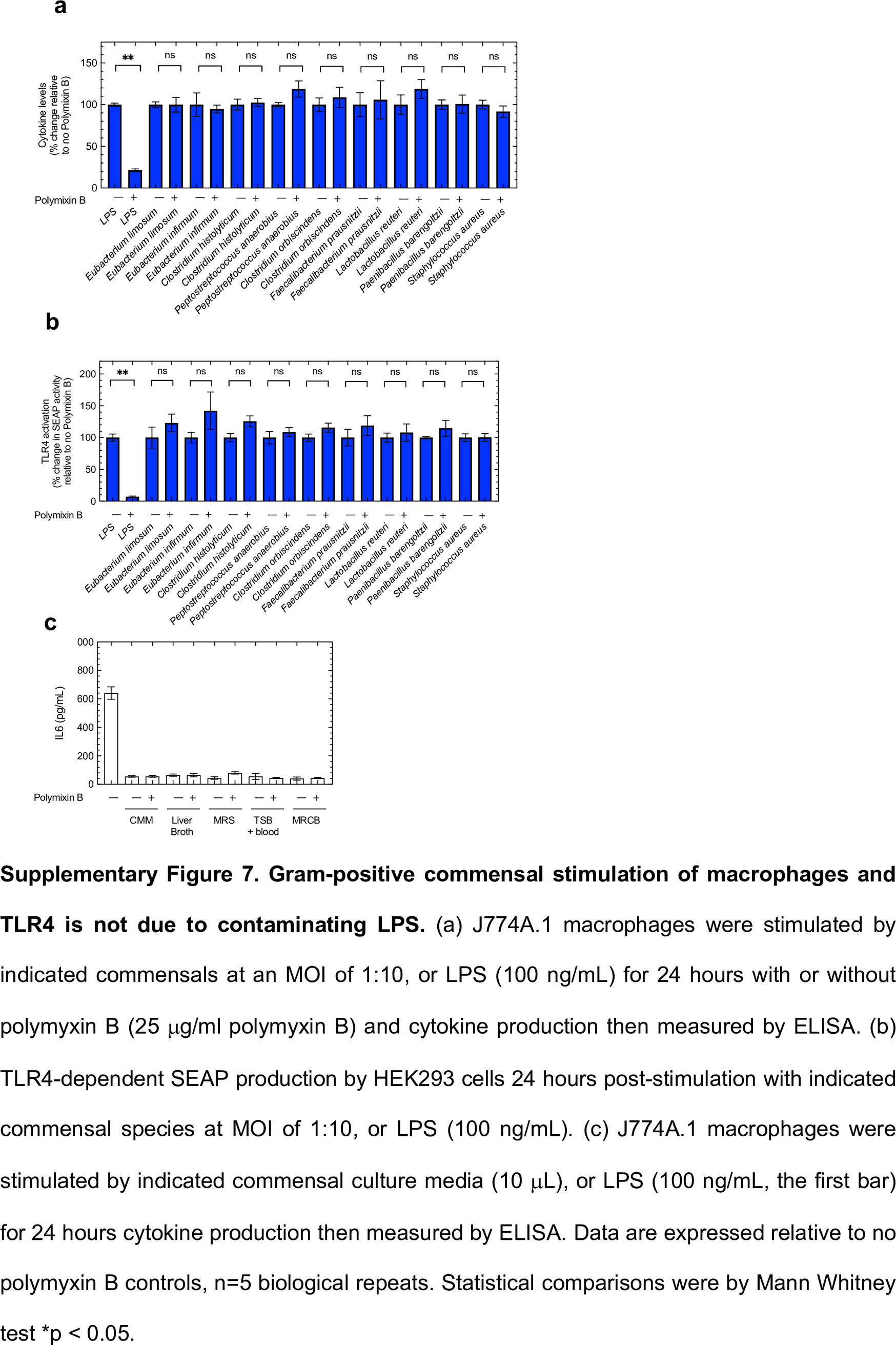

Supplement: S7 Fig — (a) J774A.1 macrophages were stimulated by indicated commensals at an MOI of 1:10, or LPS (100 ng/mL) for 24 hours with or without polymyxin B (25 μg/ml polymyxin B) and cytokine production then measured by ELISA. (b) TLR4-dependent SEAP production by HEK293 cells 24 hours post-stimulation with indicated commensal species at MOI of 1:10, or LPS (100 ng/mL). (c) J774A.1 macrophages were stimulated by indicated commensal culture media (10 μL), or LPS (100 ng/mL, the first bar) for 24 hours cytokine production then measured by ELISA. Data are expressed relative to no polymyxin B controls, n = 5 biological repeats. Statistical comparisons were by Mann Whitney test *p < 0.05, NS, not significant. (TIF) [file ppat.1009191.s007.tif]

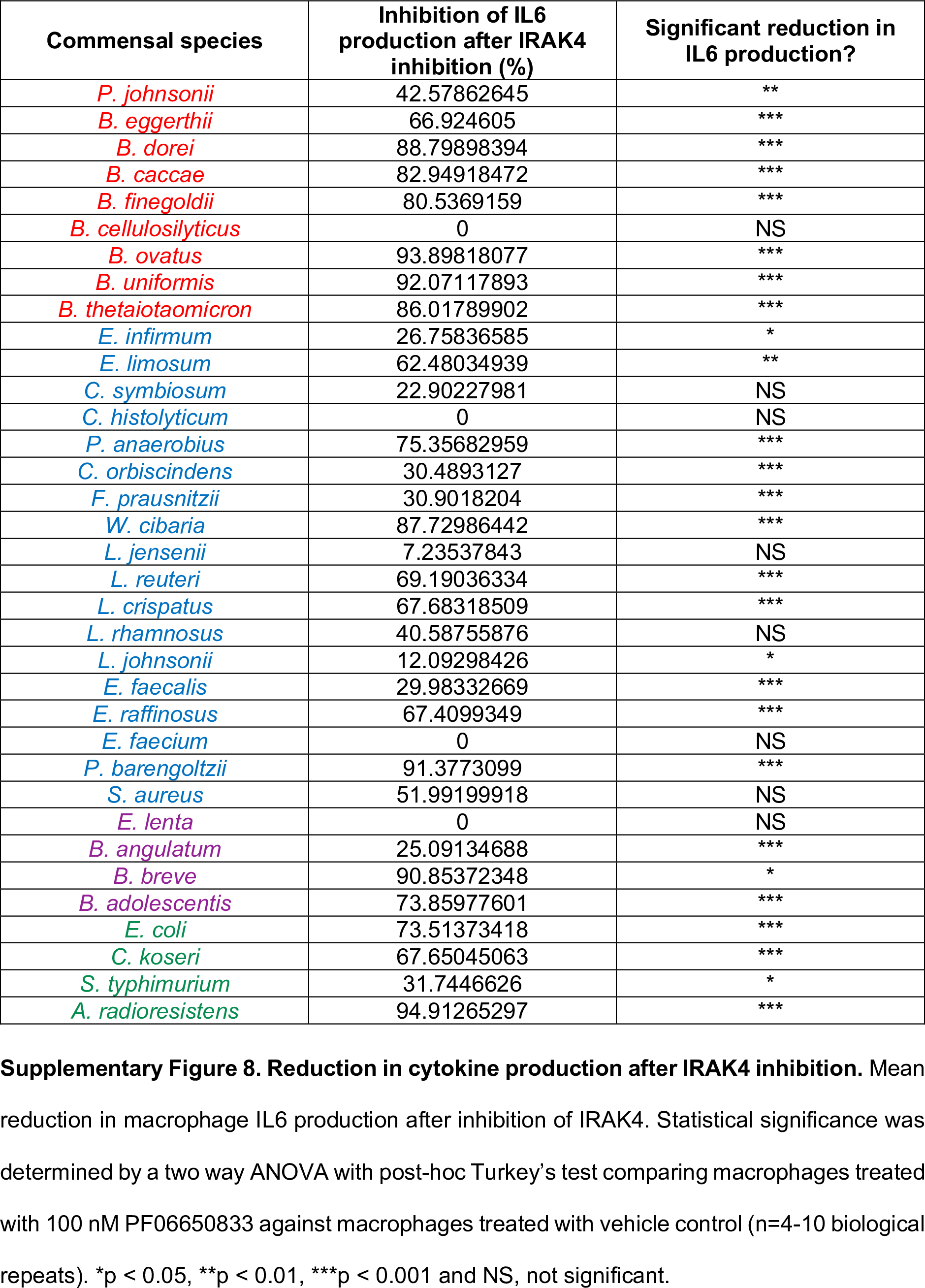

Supplement: S8 Fig — Mean reduction in macrophage IL6 production after inhibition of IRAK4. Statistical significance was determined by a two way ANOVA with post-hoc Turkey’s test comparing macrophages treated with 100 nM PF06650833 against macrophages treated with vehicle control (n = 4–10 biological repeats). *p < 0.05, **p < 0.01, ***p < 0.001 and NS, not significant. (TIF) [file ppat.1009191.s008.tif]

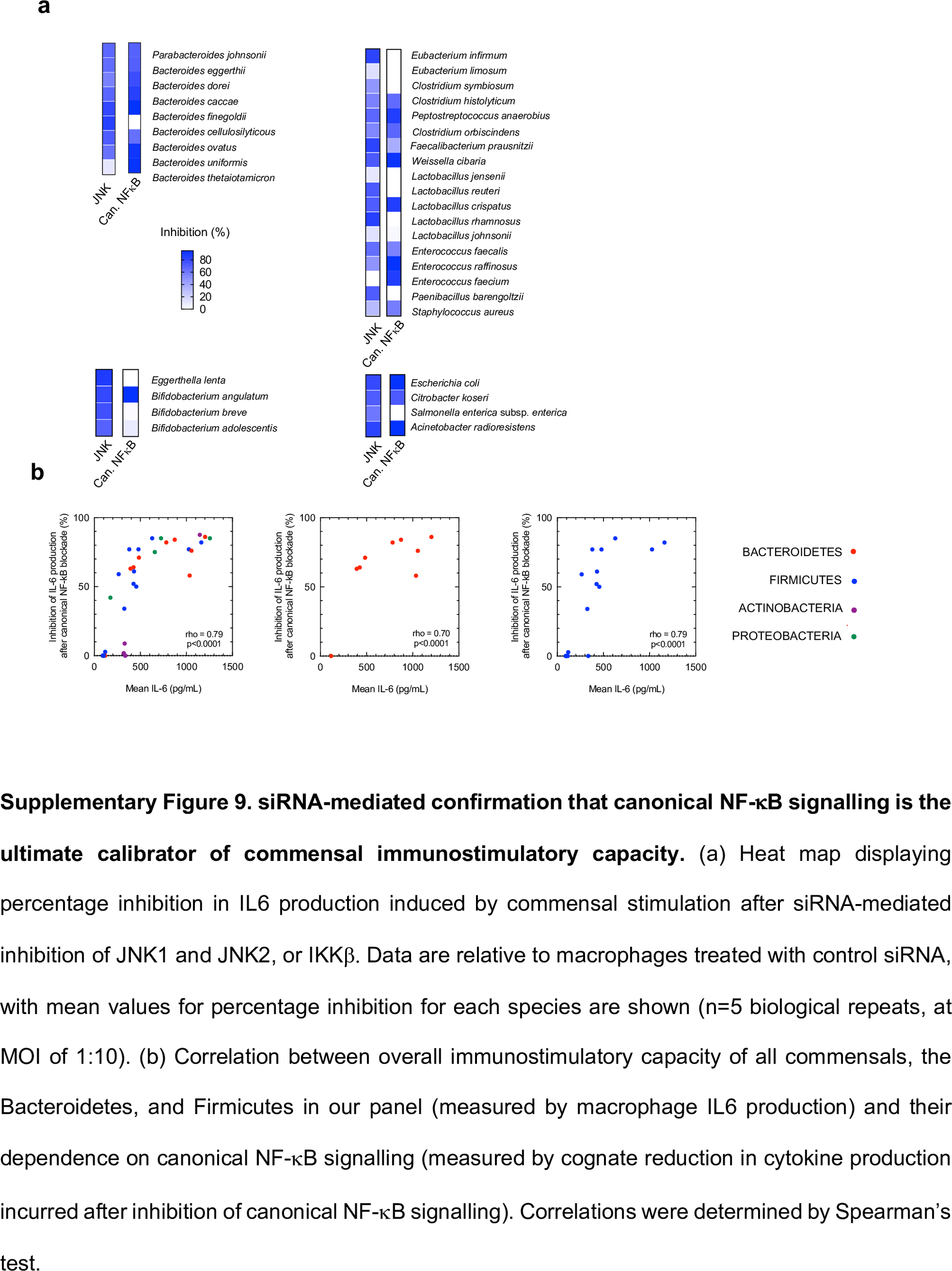

Supplement: S9 Fig — (a) Heat map displaying percentage inhibition in IL6 production induced by commensal stimulation after siRNA-mediated inhibition of JNK1 and JNK2, or IKKβ. Data are relative to macrophages treated with control siRNA, with mean values for percentage inhibition for each species are shown (n = 5 biological repeats, at MOI of 1:10). (b) Correlation between overall immunostimulatory capacity of all commensals, the Bacteroidetes, and Firmicutes in our panel (measured by macrophage IL6 production) and their dependence on canonical NF-κB signalling (measured by cognate reduction in cytokine production incurred after inhibition of canonical NF-κB signalling). Correlations were determined by Spearman’s test. (TIF) [file ppat.1009191.s009.tif]

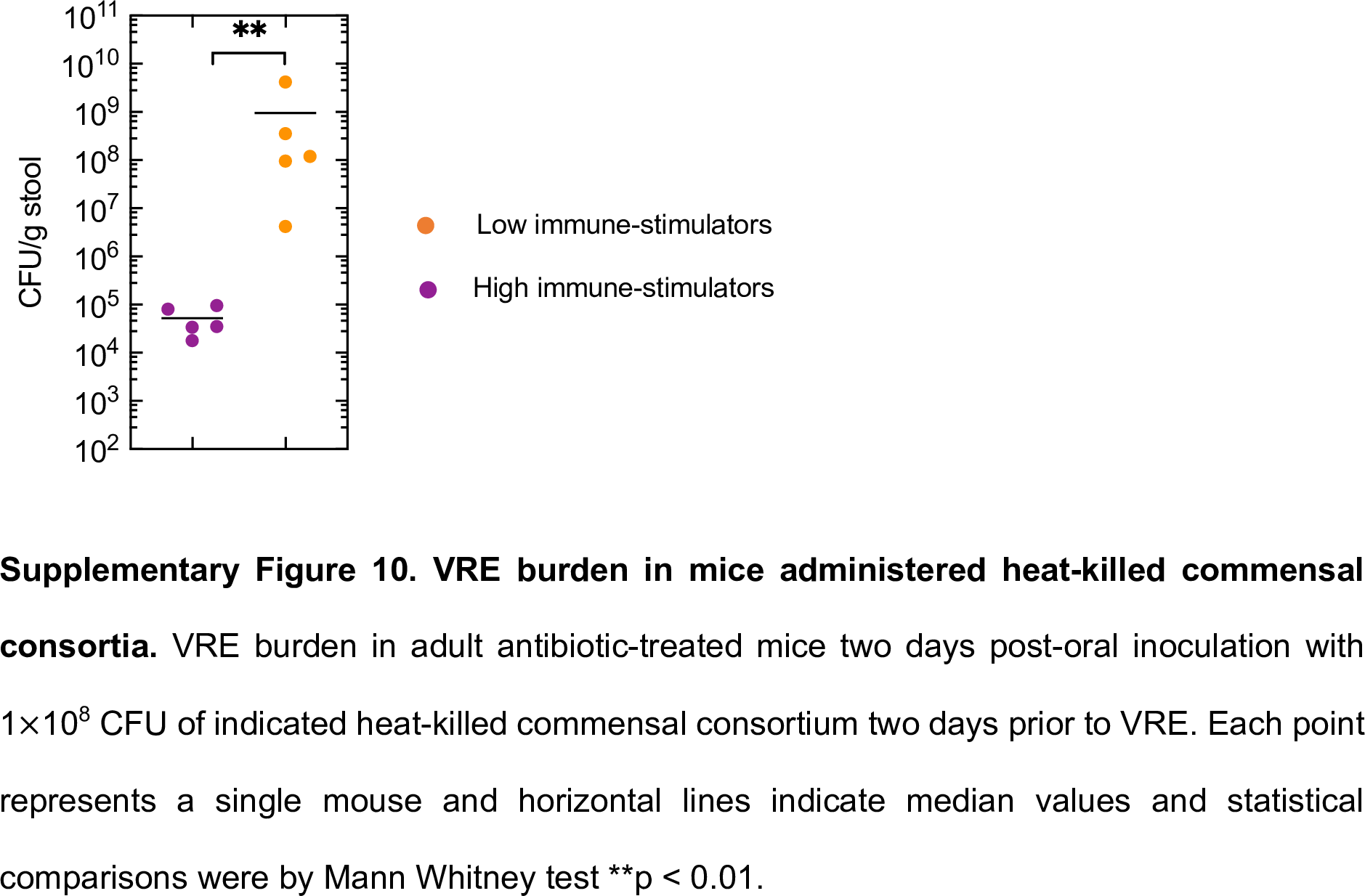

Supplement: S10 Fig — VRE burden in adult antibiotic-treated mice two days post-oral inoculation with 1×10^8^ CFU of indicated heat-killed commensal consortium two days prior to VRE. Each point represents a single mouse and horizontal lines indicate median values and statistical comparisons were by Mann Whitney test **p < 0.01. (TIF) [file ppat.1009191.s010.tif]

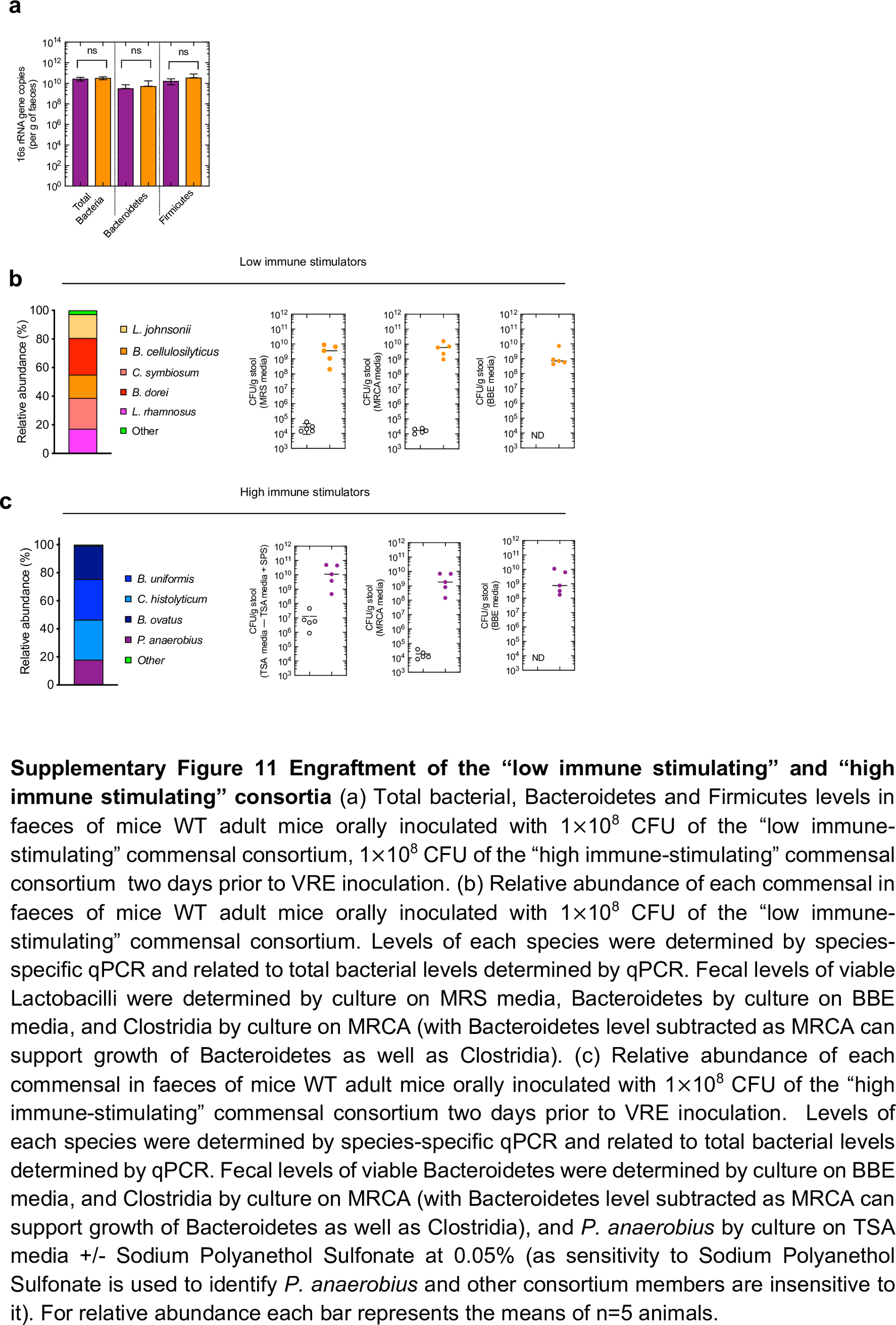

Supplement: S11 Fig — (a) Total bacterial, Bacteroidetes and Firmicutes levels in faeces of mice WT adult mice orally inoculated with 1×10^8^ CFU of the “low immune-stimulating” commensal consortium, 1×10^8^ CFU of the “high immune-stimulating” commensal consortium two days prior to VRE inoculation. (b) Relative abundance of each commensal in faeces of mice WT adult mice orally inoculated with 1×10^8^ CFU of the “low immune-stimulating” commensal consortium. Levels of each species were determined by species-specific qPCR and related to total bacterial levels determined by qPCR. Fecal levels of viable Lactobacilli were determined by culture on MRS media, Bacteroidetes by culture on BBE media, and Clostridia by culture on MRCA (with Bacteroidetes level subtracted as MRCA can support growth of Bacteroidetes as well as Clostridia). (c) Relative abundance of each commensal in faeces of WT adult mice orally inoculated with 1×10^8^ CFU of the “high immune-stimulating” commensal consortium two days prior to VRE inoculation. Levels of each species were determined by species-specific qPCR and related to total bacterial levels determined by qPCR. Fecal levels of viable Bacteroidetes were determined by culture on BBE media, and Clostridia by culture on MRCA (with Bacteroidetes level subtracted as MRCA can support growth of Bacteroidetes as well as Clostridia), and P. anaerobius by culture on TSA media +/- Sodium Polyanethol Sulfonate at 0.05% (as sensitivity to Sodium Polyanethol Sulfonate is used to identify P. anaerobius and other consortium members are insensitive to it). For relative abundance each bar represents the means of n = 5 animals. (TIF) [file ppat.1009191.s011.tif]

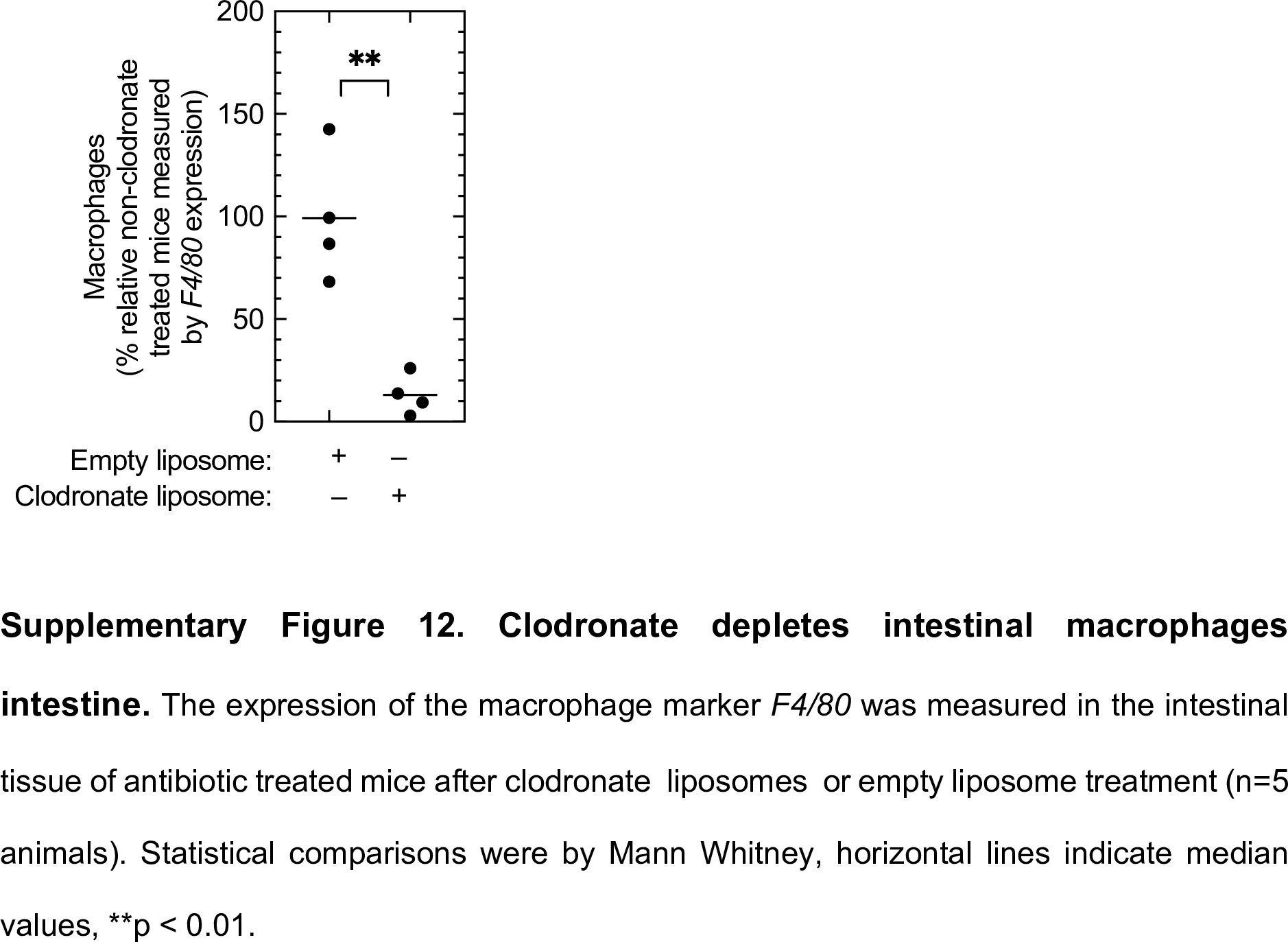

Supplement: S12 Fig — The expression of the macrophage marker F4/80 was measured in the intestinal tissue of antibiotic treated mice after clodronate liposomes or empty liposome treatment (n = 5 animals). Statistical comparisons were by Mann Whitney, horizontal lines indicate median values, **p < 0.01. (TIF) [file ppat.1009191.s012.tif]

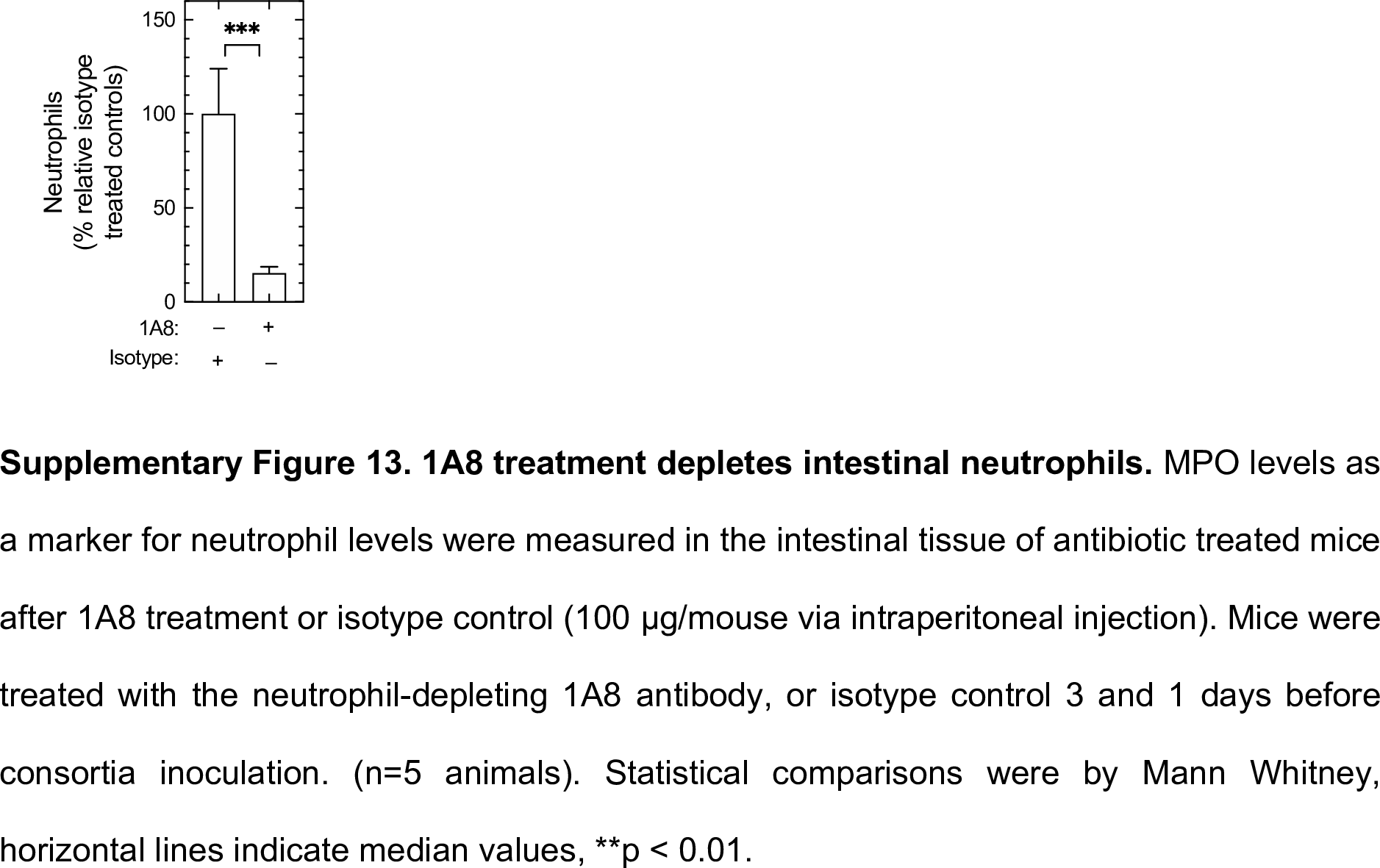

Supplement: S13 Fig — MPO levels as a marker for neutrophil levels were measured in the intestinal tissue of antibiotic treated mice after 1A8 treatment or isotype control (100 μg/mouse via intraperitoneal injection). Mice were treated with the neutrophil-depleting 1A8 antibody, or isotype control 3 and 1 days before consortia inoculation. (n = 5 animals). Statistical comparisons were by Mann Whitney, horizontal lines indicate median values, **p < 0.01. (TIF) [file ppat.1009191.s013.tif]

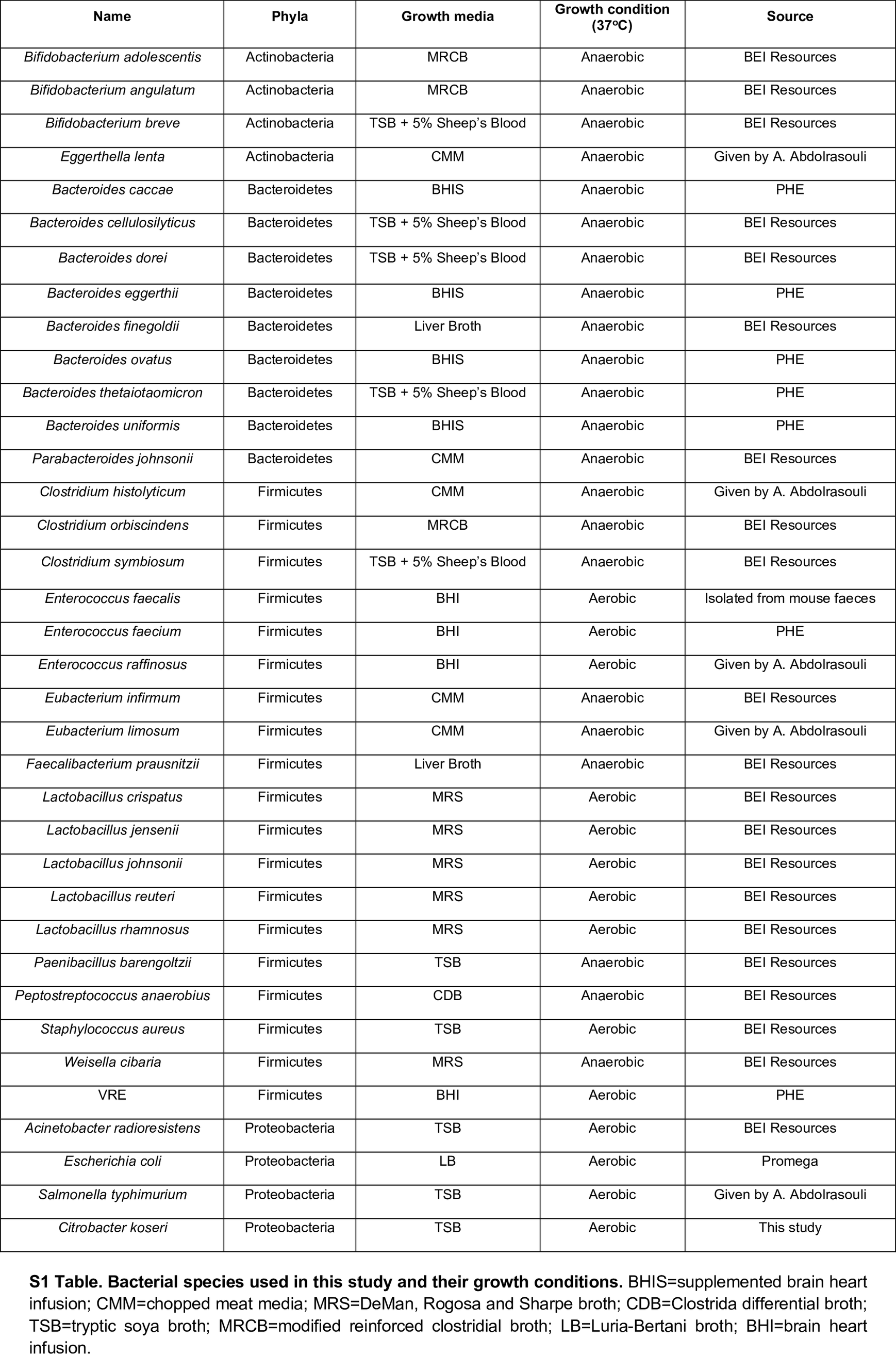

Supplement: S1 Table — BHIS = supplemented brain heart infusion; CMM = chopped meat media; MRS = DeMan, Rogosa and Sharpe broth; CDB = Clostrida differential broth; TSB = tryptic soya broth; MRCB = modified reinforced clostridial broth; LB = Luria-Bertani broth; BHI = brain heart infusion. (TIF) [file ppat.1009191.s014.tif]
